# Beta‐Arrestin‐2 Binds to Nucleolar Organizing Regions With P2X7 Receptor Stimulation and During Cell Division

**DOI:** 10.1096/fj.202601209RR

**Published:** 2026-10-08

**Authors:** Tuğba Gümüştaş, Seda Uysal, Ida Casella, Özlem Uğur, Mehmet Uğur

**Affiliations:** ^1^ Department of Pharmacology, Faculty of Medicine Ankara University Ankara Turkey; ^2^ Department of Biophysics, Faculty of Medicine Ankara University Ankara Turkey; ^3^ Dip. Farmaco Istituto Superiore di Sanita Rome Italy

## Abstract

Nonvisual *arrestins*, also known as Beta‐arrestins, are known for their diverse role in GPCR signal transduction. Here, we present evidence that Beta‐Arrestin‐2, a protein whose nuclear concentration is actively kept low, binds to Nucleolar Organizing Regions (NORs) on the metaphase chromosomes at the onset of cell division, after the disappearance of the nuclear membrane and stays bound therein in the daughter cells. After reformation of the nucleoli, it stays bound to the nucleoli until actively transported out of the reformed nucleus during interphase. This binding is unique for Beta‐Arrestin‐2, as the closely associated Beta‐Arrestin‐1 does not show this behavior at all. We also observed an intranuclear accumulation of Beta‐Arrestin‐2 after P2X7 receptor stimulation, similar to what has been described for Beta‐Arrestin‐2 after TRPV receptor stimulation. Present data identify a cyclic and well‐organized shuttling between NORs and the cytoplasm of Beta‐Arrestin‐2 during cell division, indicating a novel and unknown role for Beta‐Arrestin‐2 in cell physiology. Our data may also indicate a common intracellular signaling mechanism for a diverse group of receptors currently believed to function only as ligand‐gated channels (P2X7 and TRPV).

## Graphical abstract

Beta‐Arrestin‐2 but not Beta‐Arrestin‐1 can accumulate in the nucleolus and come in close contact with the Nucleolar Organizing Regions during two distinct processes: P2X7 receptor stimulation (A) and cell division (B). Both seem to be due to loss of nuclear membrane barrier that keeps Beta‐arrestin‐2 out of the nucleus. During cell division, Beta‐Arrestin‐2 first binds Nucleolar Organizing Regions and after nucleolus reformation, stays associated with the nucleoli. Eventually, it is pumped out of the nucleus, ending this association.

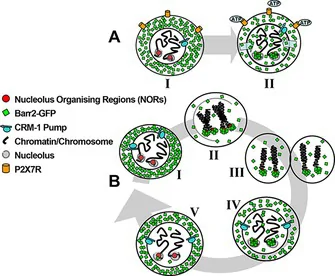

## 1 Introduction

Beta‐arrestins are well known for their important role in GPCR signal termination [1, 2]. Recently, a mass of data began to accumulate demonstrating their essential role in forming GPCR‐related complex signaling networks, distinct from the one formed by G‐proteins, with diverse and far‐reaching effects in physiological and pathophysiological processes such as gene transcription, chemotaxis, metastasis, apoptosis, and behavior [2, 3, 4]. Recent studies also suggest that Beta‐arrestins‐1 and ‐2 (Barr1 and Barr2) may interact with a massive range of intracellular proteins even in the absence of GPCR activation and imply that potential physiological functions of Beta‐arrestins may transcend their familiar role in GPCR signal transduction [5, 6, 7, 8]. However, present literature contains little direct evidence for functions other than GPCR signaling. Few examples in the literature indicate a possible role of Barr2 in cell division by regulating centrosome function [8] and also a possible role in TRPV1 receptor signaling via interacting with nucleolar proteins [5, 6].

Barr2 is a cytoplasmic protein, known to be excluded from the nucleus by active transport via CRM‐1 pump [9, 10, 11]. Thus, identifying the proteins or organelles that Barr2 can interact with in the living cells, and also understanding which physiological and physiopathological mechanisms regulate its exclusion from the nucleus are two important initial steps to uncover novel actions of Barr2. Here, we present data demonstrating that in HEK‐293 cells, Barr2 (but not Barr1) can accumulate in the Nucleolar Organizing Regions (NORs) in nucleoli in two different and unrelated conditions: (i) during cell division, in a cell‐cycle dependent cyclical way, and (ii) upon stimulation of P2X7 receptor (P2X7R) which is a ligand‐gated channel implicated in various physiological and pathophysiological processes including inflammation and tumorigenesis [12, 13, 14].

NORs are certain stretches of DNA on the acrocentric chromosomes. They form the core of the nucleoli and contain multiple repeats coding for rRNA and binding sites for various proteins, such as UBF, that are part of the rRNA transcription, processing and ribosome assembly processes [15], (For a review see [16]). In conjunction with the previous data showing the binding of Barr2 to centrosomes [8], cell cycle‐dependent nucleolar accumulation suggests a possible role for Barr2 in cell division. Accumulation of Barr2 in the nucleoli upon P2X7R stimulation, on the other hand, seems to be similar to the one observed after stimulation of the TRPV1 receptor, which is also a ligand‐gated channel. TRPV1 receptor‐induced translocation of Barr2 was proposed to play a role in the signal transduction of this receptor [5, 6]. TRPV1 and P2X7 receptors, despite the fact that being structurally distinct ligand‐gated channels, display peculiar functional similarities such as being permeable to large molecules and inducing cell blebbing. Thus, our observation that P2X7R stimulation, similarly to TRPV1 receptor stimulation, also leads to nucleolar Barr2 accumulation may suggest a role of this accumulation in P2X7R signal transduction as well.

## 2 Materials and Methods

### 2.1 Cells and Transfection

P2X7R‐expressing HEK‐293 cell clone (P2X7‐HEK) used in this study was established in our laboratory by stable transfection of HEK‐293 cells, using a rat P2X7R c‐DNA (a kind gift from Drs. Anmarie Surprenant and Alan North). Pharmacological and functional properties of these cells pertinent to P2X7R expression have previously been reported [17]. Cells expressing Renilla‐GFP‐Beta‐arrestin‐1 and 2 fusion proteins (designated as Barr1‐P2X7‐HEK and Barr2‐P2X7‐HEK, respectively) were obtained by transfecting the P2X7‐HEK clone, using a retroviral gene transfer and expression system (Clontech), as described by the manufacturer. In both transfections, cells were selected first for their antibiotic resistance (G‐418 for P2X7‐HEK and hygromycin for Barr1‐P2X7‐HEK and Barr2‐P2X7‐HEK cells). Expression of the transfected proteins was then confirmed by microscopy; the presence of specific cationic dye (YoPro‐1) uptake responses for P2X7R expression or GFP fluorescence for Renilla‐GFP‐Beta‐arrestin‐1 or 2 expression. P2X7‐HEK clone is a monoclone whereas Barr1‐P2X7‐HEK and Barr2‐P2X7‐HEK cells are polyclonal. Renilla‐GFP‐Beta‐arrestin‐1 and 2 fusion protein constructs used in this study had previously been shown to be functional in terms of their interactions with G‐protein coupled receptors [18].

### 2.2 Experimental Solutions and Protocols

Cells were grown in DMEM supplemented with 10% fetal calf serum, in a humidified atmosphere of 5% CO_2_ at 37°C. For live cell experiments, cells were seeded and grown for 48 h on cover slips and were transferred as such to the microscope chamber. Alternatively, they were grown in chamber slides for use in immunocytochemistry.

In P2X7R activation experiments, a bath solution of (in mM) Na^+^ 5, K^+^ 145, HEPES 16, pH 7.4 with no divalent ions added was used to avoid ATP chelation by divalent cations and also to limit the detrimental effects of P2X7R‐induced large intracellular ion concentration changes in the cells. In others where Barr‐GFP‐HEK cells were tracked throughout the cell division, a bath solution of (in mM) Na^+^ 125, K^+^ 5, Ca^++^ 1.5, Mg^++^ 1, HEPES 20, pH 7.4 was used. ATP concentration (1 mM) used for P2X7R stimulation is in accordance with what has been published for this receptor [19]. In dye‐uptake experiments, ToPro‐3 (Thermo Fisher Scientific, Cat. No: R37113) was added to the bath solution during the ATP application at a concentration of 2 μM.

For LMB experiments, cells were incubated overnight in serum‐free DMEM either with the corresponding amount of vehicle (ethanol) or with LMB (20 ng/mL, Sigma‐Aldrich, Cat. No: L2913).

### 2.3 Immunocytochemistry

Method described in [20] was used with minor modifications. Cells, grown in chamber slides, were washed four times with PBS at room temperature, fixed with 2% (wt/vol) paraformaldehyde in PBS for 20 min and permeabilized with 0.1% (wt/vol) Triton X‐100 in PBS for 15 min. After incubation for 15 min in the blocking buffer (1% normal goat serum, 0.2% [vol/vol] Triton X‐100 in PBS), cells were incubated for 1 h at room temperature in 0.1% (wt/vol) BSA in PBS with the primary antibodies (Santa Cruz Biotechnology, Cat. Nos: Sc‐166 001, Sc‐55 486 and Sc‐13 125 for anti‐fibrillarin, anti‐nucleolin and anti‐UBF, respectively) at a 1:50 dilution. After washing four times with 0.1% BSA in PBS, cells were incubated for 1 h at room temperature with Alexa Fluor 594‐labeled anti‐rabbit antibody (Jackson ImmunoResearch, Cat. No: 111–585‐045) at a 1:100 dilution. Staining of fixed cells with Sytox Orange (Thermo Fisher Scientific, Cat. No: S‐11368) was conducted using the dye at a concentration of 50 nM.

### 2.4 Confocal Microscopy

Leica SP‐5 laser scanning confocal microscope was used for the imaging of both the live and fixed cells. We used either a 63X, NA 1.2 oil immersion lens (for live cell experiments) or a 40X, NA 1 water immersion lens (for fixed preparations). All the preparations were imaged as multiple z‐stacks (stack numbers are variable among different experiments). GFP fluorescence was imaged by using 488 nm laser excitation and by collecting emission between 510 and 585 nm. For Alexa Fluor 594 we used 594 nm excitation and collected emission between 640 and 700 nm. For excitation of Sytox Orange we used 543 nm and for ToPro‐3 we used 633 nm lasers and collected emissions between 600–670 nm and 700–770 nm, respectively. For imaging double stained preparations, we used sequential imaging to avoid fluorescence bleed‐through. For fixed preparations, 2–3 line averaging was employed. In live cell imaging experiments, laser power was kept at the possible minimum value to avoid cell damage. In the latter, cover slips containing the cells were placed at the base of a special chamber which holds the bath solution. A microscope stage heater with a thermostat (Leica) is used to keep the cells at 37°C. The chamber was covered with a lid to avoid evaporation and liquid levels were checked for liquid loss after each experiment was concluded.

### 2.5 Environment‐Controlled Long Term Microscopy Experiments

We used a Leica‐MICA (Leica Microsystems, Germany) environment‐controlled microscopy system. We imaged Barr2‐GFP‐expressing HEK‐293 cells grown in DMEM containing 10% calf serum on an Ibidi imaging and culturing chamber with a coverslip bottom (Ibidi GmbH, Germany). Imaging was performed in the same medium. We observed and imaged the cells for 5 h in a closed 5% CO_2_, 37°C environment, using an integrated upright fluorescent microscope with a 63X water immersion lens. Sequential images from multiple adjacent fields of view were taken by the system every 15 or 30 min for 5 h, each containing 25 z‐planes. Images were stitched together automatically by MICA software.

## 3 Results

### 3.1 P2X7R Activation Triggers Accumulation of Barr2‐GFP Into Nucleolus‐Like Structures

In HEK‐293 cells co‐expressing Beta‐arrestin‐2‐rGFP (Barr2‐GFP) and P2X7R (Barr2‐P2X7‐HEK cells), Barr2‐GFP was mostly cytoplasmic and effectively excluded from the nuclei, as previously reported [9, 10]. Using a confocal microscope, we observed that ATP stimulation led to a rapid increase in intranuclear concentration and granular aggregation of Barr2‐GFP in approximately 10% of the cells. (Cumulatively for 3529 cells from 29 independent experiments 10.9% ± 1.2%, Figure 1A, Movie S1). Barr2‐GFP accumulation response in Barr2‐P2X7‐HEK cells started approximately 5 min after the addition of ATP, and the number of responding cells increased with time. Once started, the response of the individual cells was rapid and reached completion within 1 or 2 min (This can best be seen in Movie S1). When the living Barr2‐P2X7‐HEK cells were tracked and the ones responding to P2X7R stimulation were examined by using overlapped fluorescence and DIC images, the accumulation appeared to be within the nucleoli inside the intact nuclei (Figure 2A). DNA staining of the cells with ToPro‐3, by utilizing the well‐known phenomenon of cationic P2X7R‐induced dye permeation [17], clearly showed that the Barr2‐GFP accumulation was in close proximity with the nuclear chromatin (Figure 2B). ATP stimulation also caused a fast and dramatic increase in intranuclear Beta‐arrestin‐1‐rGFP (Barr1‐GFP) concentration in some of the cells co‐expressing P2X7R and Barr1‐GFP. However, unlike what is seen with Barr2‐GFP, this increase did not lead to any granular accumulation of Barr1‐GFP within the nucleus (Figure 1B; Movie S2). It is important to point out that the cells identified as responding to the P2X7R stimulation were not dividing cells; their nuclear membranes were intact as can be seen in Figure 2A,B. Moreover, they do not show any intranuclear accumulation before the P2X7R stimulation (Figure 1A, Movie S1). The cells were tracked for a period of 30 min after ATP application and those cells showed a Barr2‐GFP accumulation without dividing, counted as responding cells. These points are important in order to distinguish the P2X7R‐induced Barr2‐GFP accumulation response from the accumulation observed in non‐stimulated cells and cells that do not express P2X7R, which will be described below.

**FIGURE 1:**
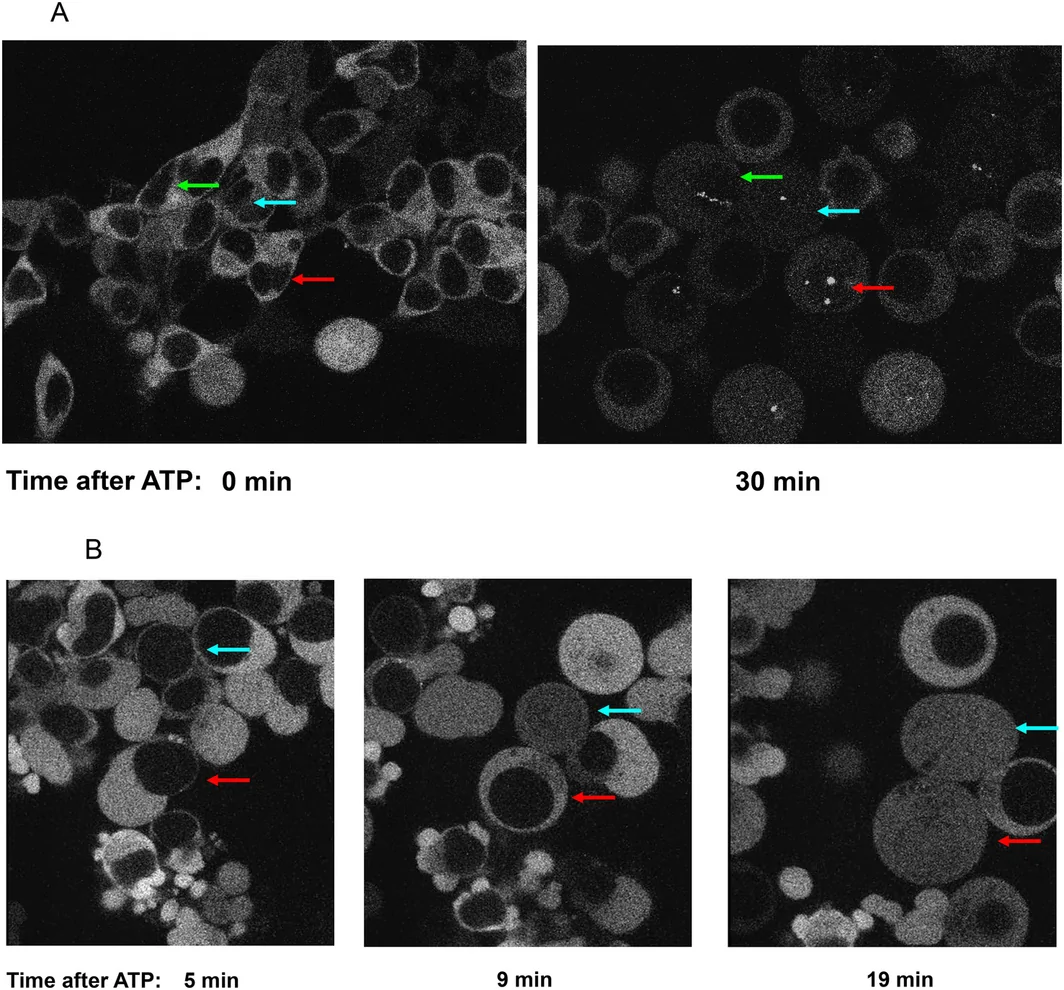
ATP application to P2X7 receptor expressing HEK‐293 cells rapidly increases the nucleoplasmic concentration of both Barr2‐GFP and Barr1‐GFP, but only Barr2‐GFP shows granular nuclear accumulation. Live cells co‐expressing P2X7 receptor and Barr‐GFP were stimulated with 1 mM ATP at 0 min. After ATP application, consecutive z‐stack images were obtained for 20–30 min. Images show single slices from the stacks captured at indicated times after ATP application. (A) Three Barr2‐GFP expressing cells are individually labeled with differently colored arrows. At 30 min, all three cells begin to show clear granular accumulation spots. Images are representative of 15 experiments on 12 different days. (B) Two Barr1‐GFP expressing cells are individually labeled with differently colored arrows. Although the nucleoplasmic Barr1‐GFP concentration increases dramatically at 9 and 19 min in blue and red‐labeled cells, respectively, no granular accumulation of Barr1‐GFP can be observed (As stimulation of P2X7 receptor with ATP causes intensive blebbing and cell movement, it is difficult to follow individual cells in between still photos. Please refer to the Movies S1 and S2 for the whole sequence of images in which individual cells can be tracked more easily).

**FIGURE 2:**
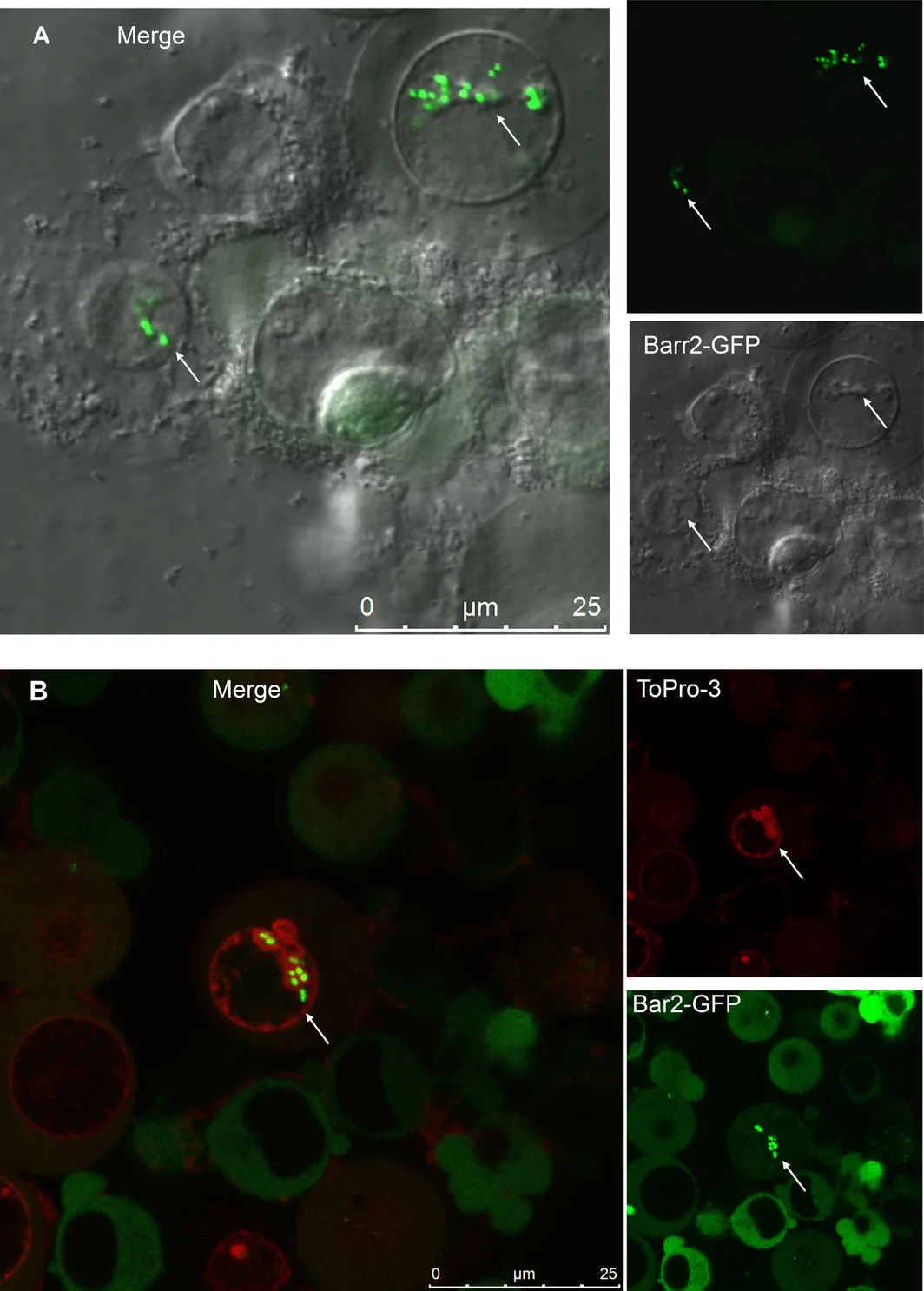
P2X7 receptor stimulation induces accumulation of Barr2‐GFP into the nucleoli. Live cells expressing Barr2‐GFP and P2X7 receptor were treated with ATP (1 mM). (A) DIC (gray) and Barr2‐GFP fluorescence (green) images, as well as a merged image of the two are presented. (B) A different experiment in which the genetic material of the nucleus and nucleolus was stained by DNA dye ToPro‐3, taking advantage of the membrane permeabilizing effect for cationic large molecules of P2X7R stimulation by ATP (1 mM) is shown. ToPro‐3 (red) and Barr2‐GFP (green) fluorescence images as well as a merged image of the two are presented in (B). Images in both (A) and (B) were taken after the cells were tracked individually through a 20‐min experiment and were positively identified to respond to agonist application by forming granular Barr2‐GFP accumulation in their nucleus. Note that in both images, granular Barr2‐GFP accumulation appears to be in nucleolus‐like structures (arrows) inside the nucleus. Also note that the cell does not present any metaphase chromosome staining with ToPro‐3 and thus is non‐dividing. Images are representative of 16 cells from 5 different experiments in 4 different days for A, and 7 cells in 3 different experiments in 2 different days for B.

The phenomenon described above, that is, accumulation of Barr2‐GFP within the nucleoli‐like structures following the P2X7R stimulation, has not been reported before. In addition to the fact that Barr2‐P2X7‐HEK cells, used in this study are derived from a P2X7R‐expressing cell clone which was already extensively established in a previous study [17], following are the findings clearly indicating that the ATP response seen in Barr2‐P2X7‐HEK cells is indeed due to P2X7R stimulation: (i) The ATP effect could only be observed in P2X7R‐expressing cells: Cells that express Barr2‐GFP but not the P2X7R did not show any detectable ATP‐induced accumulation (5 parallel experiments with P2X7R‐expressing cells, on 5 different days, 543 cells); (ii) UTP (1 mM) and ADP (1 mM), which are agonists for most P2Y receptors but not for P2X7R, did not trigger any such accumulation in Barr2‐P2X7‐HEK cells (4 experiments done in parallel with 1 mM ATP application, on 4 different days 0.2% ± 0.1% and 0.2% ± 0.2% respectively for UTP and ADP, 496–917 cells); and (iii) BzATP, an agonist with a greater potency at P2X7R compared to ATP, induced the Barr2‐GFP accumulation when applied at 5 μM concentration, while ATP at the same concentration had no effect on Barr2‐P2X7‐HEK cells, 3 experiments on 3 different days, (0.4% ± 0.2% and 9.8% ± 1.6% for ATP and BzATP respectively, 915–1013 cells).

### 3.2 Beta‐Arrestin‐2‐GFP Also Accumulates Into Granular Structures Within the Nuclei of Non‐Stimulated and Non‐P2X7R Transfected HEK‐293 Cells

In addition to the P2X7R‐induced accumulation presented above, we noticed a significant background related to this phenomenon: A small but significant number of Barr2‐P2X7‐HEK cells displayed a visually similar Barr2‐GFP accumulation within their nuclei without any P2X7R stimulation. Furthermore, a similar accumulation was also observable in a subset of HEK‐293 cells that expressed Barr2‐GFP but not the P2X7R (5.3% ± 0.9% of 345 cells from 3 experiments) (Figure 3A). The Barr2‐GFP accumulation in these cells appeared to be static for the 30 min duration. Examination of the overlapped fluorescence and DIC images suggested that these spots of Barr2‐GFP accumulation might also be within the nucleoli, as in the case of ATP stimulation. As can be seen in Figure 3B, in Barr1‐GFP expressing cells no such intranuclear accumulation was observed. In order to gain insight as to the exact nature of this accumulation process, we further investigated this spontaneous accumulation in Barr2‐GFP expressing HEK‐293 cells that do not express P2X7R (Barr2‐HEK cells). It is important to note that we also observed peri/extra‐nuclear granular accumulation of Barr2‐GFP in a significant number of cells. They are smaller in appearance than the intranuclear accumulations (Figure 3A). These structures are likely to be Barr2‐GFP accumulations on centrosomes as reported earlier [8].

**FIGURE 3:**
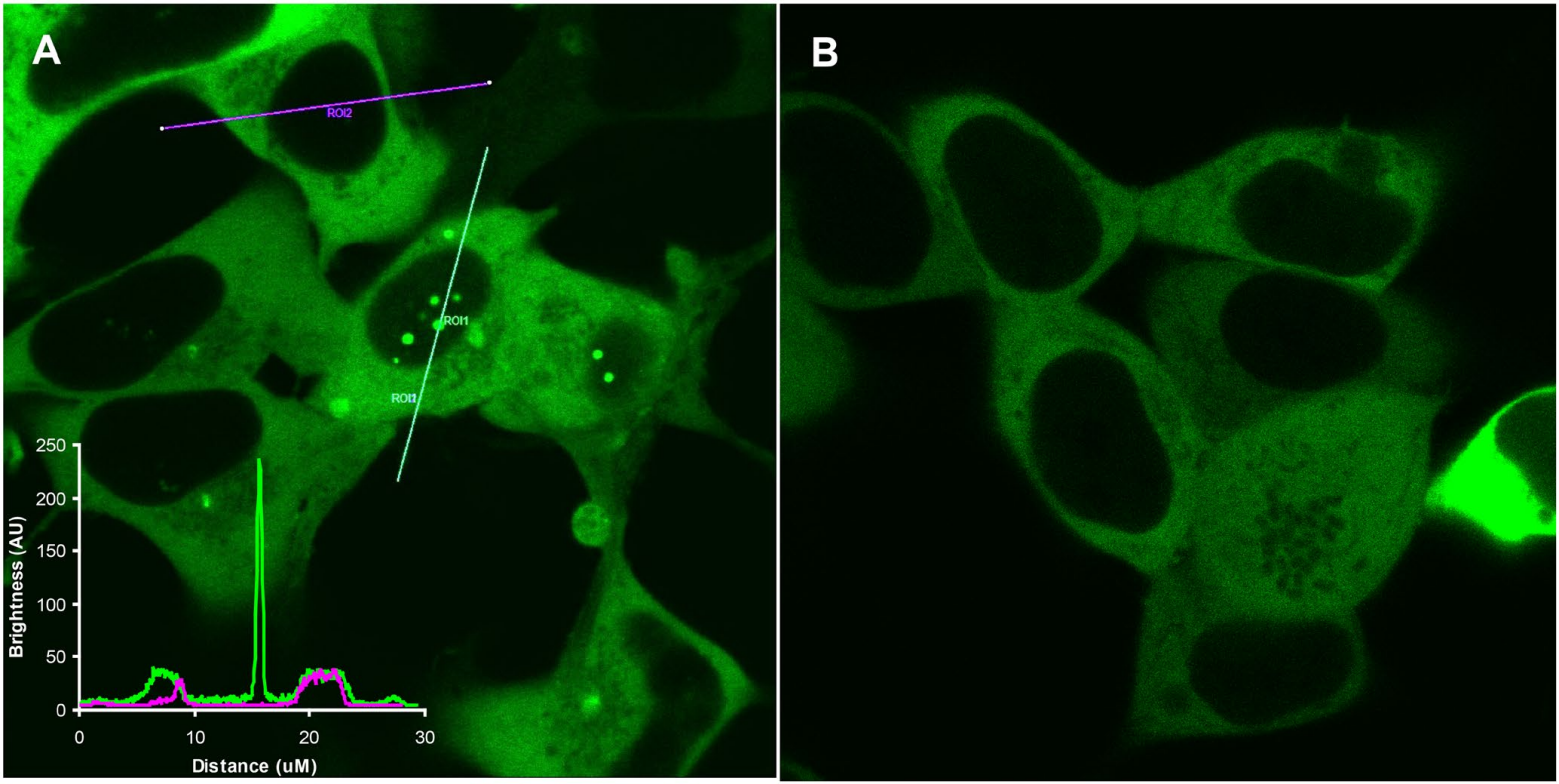
Barr2‐GFP, but not Barr1‐GFP, accumulates in granular structures within a sub‐group of HEK‐293 cells. Live cells expressing Barr2‐GFP (A) or Barr1‐GFP (B) were imaged. (A) A large proportion of the Barr2‐GFP expressing cells have a significantly low intranuclear Barr2‐GFP signal compared to the cytoplasm. Only a small group of cells display granular accumulation of Barr2‐GFP in the nucleus. Inset shows the line‐scan data along the lines drawn, going through cells with (green) or without (magenta) granular Barr2‐GFP accumulation. (B) Even though the intracellular distribution of Barr1‐GFP and Barr2‐GFP is similar, there is no sign of granular accumulation in any of the Barr1‐GFP cells. All images show a single optical slice, Lines are 30 μM long for A. Scale is the same for A and B. Note that Barr2‐GFP signal can reach levels several folds higher within the granular structures compared to the signal in the nuclei and the cytoplasm (inset green lines).

### 3.3 Granular Structures in Which Barr2‐GFP Accumulates Are the Nucleoli

In order to verify the assumption that the intranuclear structures where Barr2‐GFP accumulates are actually the nucleoli, we performed immunofluorescent labeling in unstimulated Barr2‐HEK cells. We used primary antibodies against three different nucleolar proteins (Anti‐Fibrillarin, anti‐Nucleolin, or anti‐UBF antibodies) and an Alexa Fluor 594‐conjugated secondary antibody (Figure 4A–C, respectively). Each antibody strongly stained small structures within the nuclei, which we concluded to be the nucleoli. Nucleoli staining obtained by all of these anti‐nucleolar antibodies showed strong spatial association with Barr2‐GFP signal in cells that had granular Barr2‐GFP accumulation in their nuclei (Figure 4). These results strongly indicate that Barr2‐GFP indeed accumulates within the nucleoli in Barr2‐HEK cells. However, examination of GFP and Alexa Fluor 594 signals at higher resolution revealed that anti‐Fibrillarin and anti‐Nucleolin staining did not always perfectly colocalize with the GFP signal: In most of the cells, antibody fluorescence and the GFP signal, although always in close spatial association, clearly showed different micro‐patterns (Figure 4D). In contrast, for the anti‐UBF antibody, the colocalization of the Alexa Fluor 594 and GFP signals was always perfect within the optical resolution of the microscope (Figure 8). All cells that we could detect to show intranuclear granular Barr2‐GFP accumulation displayed colocalization with Anti‐Fibrillarin, anti‐Nucleolin, or anti‐UBF antibody staining, without exception.

**FIGURE 4:**
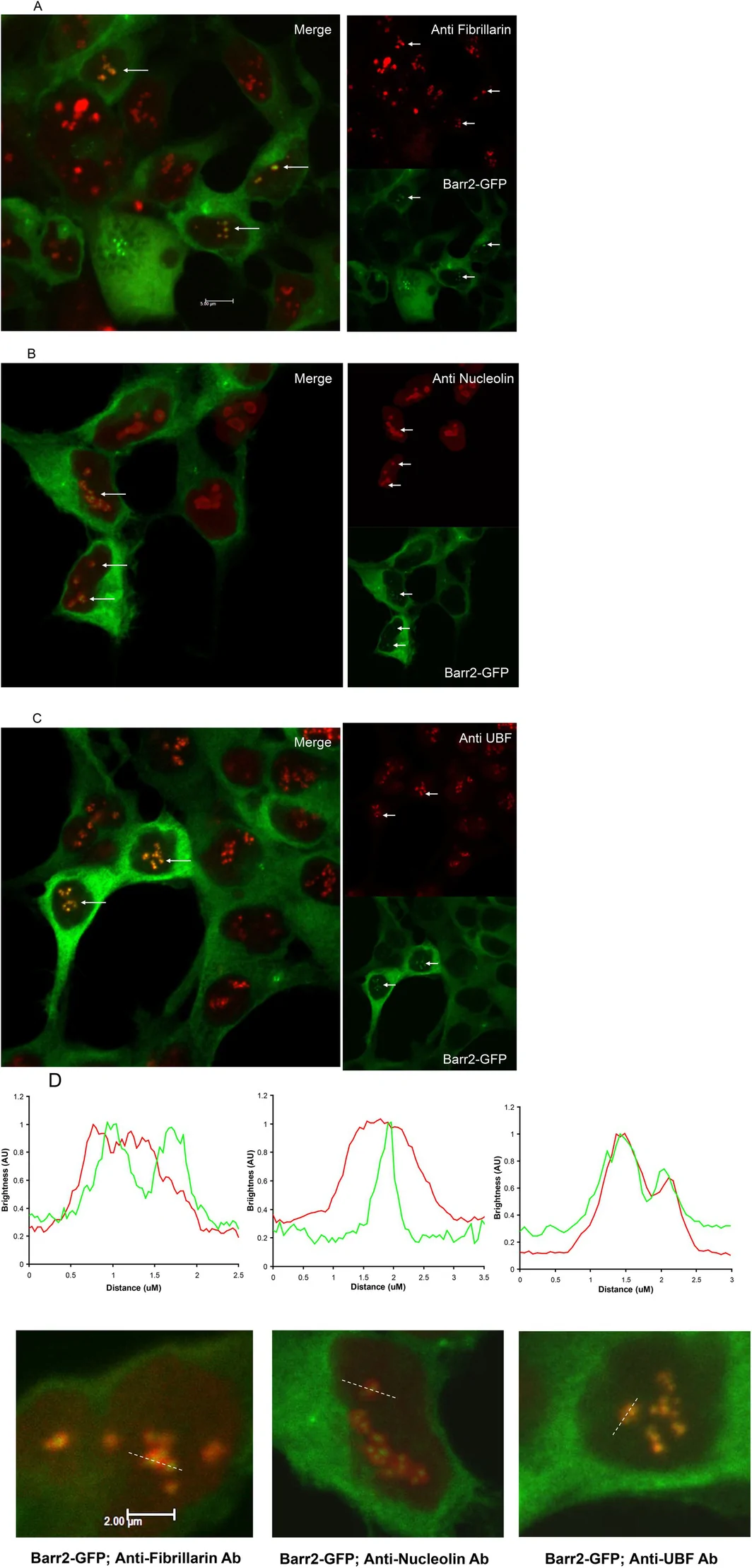
Staining with anti‐Fibrillarin, anti‐Nucleolin, or anti‐UBF antibodies reveals that Barr2‐GFP accumulates in the nucleoli. Cells expressing Barr2‐GFP were fixed and stained with anti‐Fibrillarin (A), anti‐Nucleolin (B), or anti‐UBF (C) Abs and imaged. Images of Ab staining (red) and Barr2‐GFP (green, right panels), as well as a merged image (left panel), are presented. Arrows indicate examples of structures that show both strong Barr2‐GFP signal and anti‐Fibrillarin (A), anti‐Nucleolin (B), or anti‐UBF (C) staining. Notice that in all three preparations, all cells show a granular anti‐nucleolar protein staining, whereas only a few show granular Barr2‐GFP accumulation. Also note that, except for the cell without a discernable nucleus (lower part of the image in A), all granular Barr2‐GFP signal within the nuclei is colocalized with the anti‐nucleolar protein staining. Images are obtained by merging two consecutive optical slices. Scale is indicated on the merged images. Images are representative of 10, 14, or 22 fields examined in 3, 5, or 9 independent experiments for fibrillarin, nucleolin, or UBF stainings, respectively. (D) Cells were stained with Abs as indicated. Upper panels: Line‐scan data taken along the lines shown on the corresponding microphotographs. Lower panels: Merged images of the two fluorescence channels. Data in upper panels are normalized to their corresponding peak signal values. Red traces show signals from anti‐Fibrillarin, anti‐Nucleolin, or anti–UBF staining, as indicated. Green traces show Barr2‐GFP signal. Notice that anti‐UBF staining shows the best colocalization with the GFP signal on the sub‐micron scale. Images show single optical slices. For colocalization of Barr2‐GFP and nucleolar proteins in LMB treated cells, see Figures S2A–C and S3. For UBF and Barr2‐GFP colocalization in dividing cells, see Figure 8 and Movie S4. Lines across the cells are 2.5, 3.5, and 3.0 μM long for anti‐Fibrillarin, anti‐Nucleolin, and anti‐UBF staining, respectively.

Similar to Barr2‐GFP, Barr1‐GFP was also excluded from the nucleus when expressed in HEK‐293 cells. However, in stark contrast to Barr2‐GFP, it did not show any granular accumulation within the nucleus (Figure 3). It should be noted that the distribution pattern of Barr1 is similar to what has been reported by Groarke et al. [21] and Song et al. [22]. However, it indicates a considerably larger difference between the cytoplasm and the nucleus than what was presented in [9, 11]. This point is discussed in the final section. As expected, no association between Barr1‐GFP and Alexa Fluor 594 signals could be observed in Fibrillarin‐, Nucleolin‐, or UBF‐stained Barr1‐GFP expressing cells (Data not shown).

### 3.4 Inhibition of the CRM‐1 Pump by Leptomycin‐B Increases the Accumulation of Barr2‐GFP Within the Nucleoli

After assessing that Barr2‐GFP indeed accumulates into the nucleoli of unstimulated cells, we investigated the mechanism of this accumulation. We postulated that Barr2‐GFP might have an intrinsic affinity for the nucleoli and the cells might be impeding this accumulation by keeping the concentration of Barr2‐GFP low within the nucleus via the CRM‐1 pump. To test this assumption, we used Leptomycin‐B (LMB) to inhibit the CRM‐1‐dependent nuclear export (NES‐pump). This pump complex is known to export proteins that contain a specific sequence called Nuclear Export Sequence (NES) out of the nucleus. Barr2 (but not Barr1) contains a NES and thus is exported from the nucleus via this pump system [9, 11]. We observed that, compared to the vehicle control group, LMB‐treated Barr2‐HEK cells showed a clear increase in diffuse intranuclear Barr2‐GFP signal (Figure 5A,C). The number of cells containing Barr2‐GFP granules within the nuclei was also increased dramatically (Figure 5A,D). As expected, neither a significant change in the level of the diffuse intranuclear Barr1‐GFP signal nor any granular intranuclear accumulation occurred in LMB‐treated Barr1‐HEK cells (Figure 5B,C). On the contrary, when examined with higher sensitivity, Barr1‐GFP protein in the nucleus appeared to be excluded from the nucleoli‐like small structures (Figure S1). This exclusion was evident in both the LMB‐treated and LMB‐untreated Barr1‐HEK cells.

**FIGURE 5:**
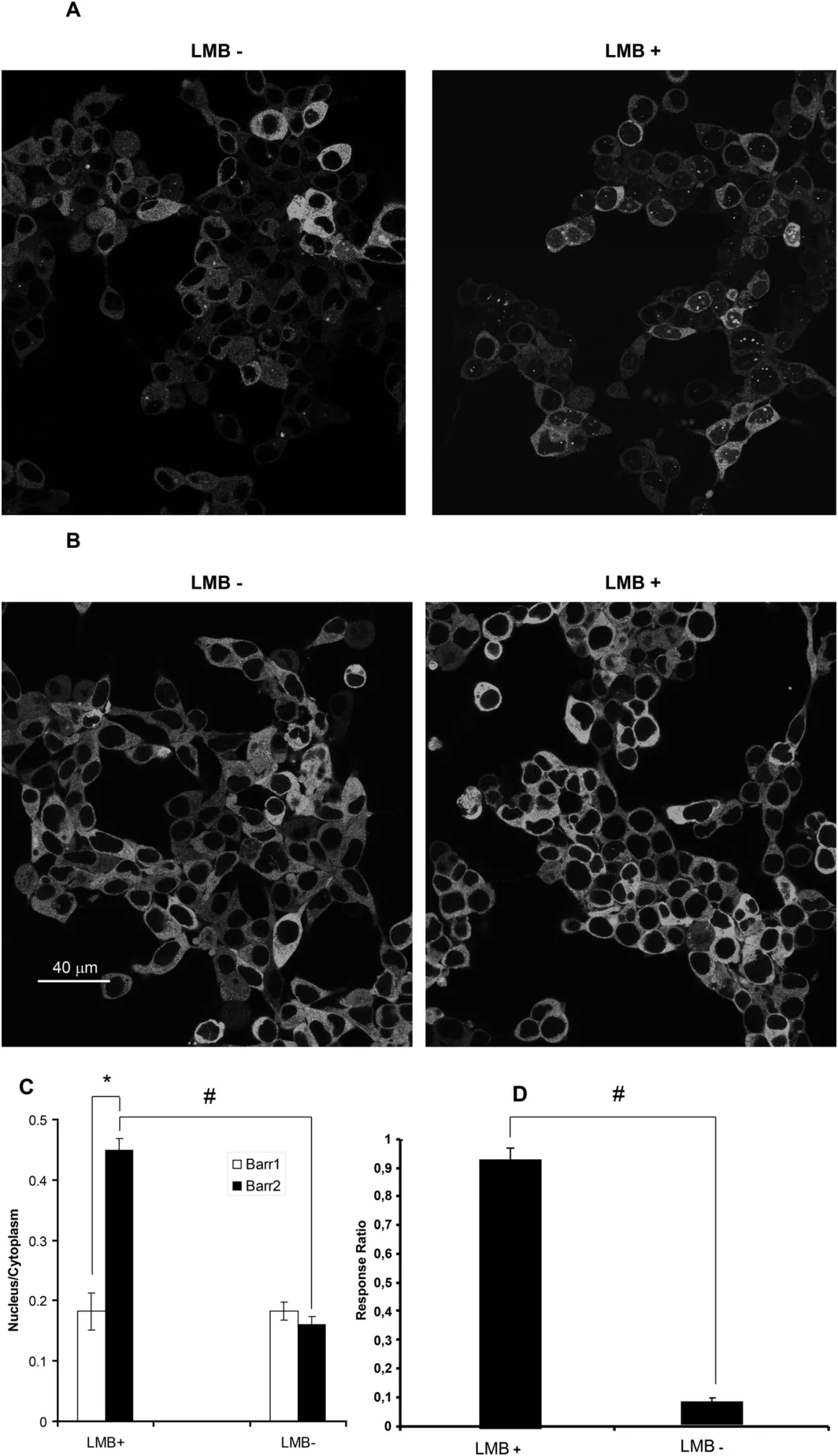
LMB increases the granular accumulation of Barr2‐GFP within the nuclei. Live cells expressing Barr2‐GFP (A) or Barr1‐GFP (B) were imaged following the overnight incubation with LMB (LMB +) or vehicle (LMB ‐). The effect of LMB treatment on the nucleoplasmic/cytoplasmic ratio of diffuse GFP signal (C) and on the ratio of the cells with nuclear granular accumulation to the total number of cells (D) is shown (only the results for the Barr2‐GFP cells are shown in D as no granular accumulation is detectable for Barr1‐GFP). Compared to the vehicle treatment, overnight LMB treatment significantly increases both the nucleoplasmic diffuse Barr2‐GFP concentration (A and C) and the number and intensity of granular Barr2‐GFP accumulation within the nuclei (A and D). LMB does not increase the nucleoplasmic concentration of Barr1‐GFP (B and C). There is no visible granular accumulation in control (LMB ‐) or LMB‐treated (LMB +) Barr1‐GFP expressing cells (B). Images show single optical slices. Scale is the same for all images in A and B and indicated on the figure. See also Figure S1 for close‐up images. Bar graphs show mean ± SEM of data obtained simultaneously in 4 independent experiments. Measurements are taken from 50 to 70 (Barr2‐GFP) or 80–100 (Barr1‐GFP) cells for each group per experiment. The difference between the untreated (LMB ‐) Barr1 and Barr2‐GFP cells and also between treated (LMB +) and untreated Barr1‐GFP cells is statistically not significant; symbols * and # indicate *p* < 0.05 (C and D).

In LMB‐treated cells, like in untreated cells, granular Barr2‐GFP signal was colocalized with Fibrillarin, Nucleolin, or UBF staining, indicating that upon LMB treatment Barr2‐GFP accumulates into the nucleoli (Figure S2). In contrast, Barr1‐GFP did not show any association with Fibrillarin or Nucleolin staining both in LMB‐treated cells and in their respective controls as expected (data not shown). Similar to what was observed in untreated cells, UBF fluorescence and Barr2‐GFP displayed the best match in the micro‐scale. Anti‐Fibrillarin and anti‐Nucleolin staining, although closely correlated with Barr2‐GFP signal, showed subtle differences of distribution in smaller scale (compare Figure 4 with Figures S2 and S3). Interestingly, in some LMB‐treated cells anti‐Fibrillarin or anti‐UBF staining showed an unusual “pairing” pattern with the Barr2‐GFP signal. We also observed homogenous spots of Barr2‐GFP signal within the nuclei with no nucleolar antibody staining in LMB‐treated cells (Figures S2 and S3). None of these staining patterns were observable in untreated cells suggesting that they might be a consequence of LMB treatment. They may be linked to the incipient nucleoli and prenucleolar bodies (PNBs), which were arrested at an early stage of nucleolus reformation following the cell division, an effect ascribed to LMB [23].

### 3.5 Barr2‐GFP Accumulates Within Granular Structures Associated With Chromosomes in Dividing Barr2‐HEK Cells

We additionally observed that, within the Barr2‐HEK cells that displayed the spontaneous granular GFP signal, a subgroup appeared to be round with no discernible nucleus (Figure 6). Within these cells, Barr2‐GFP was homogenously distributed except for the small granular structures where Barr2‐GFP signal reached high levels (15.6 ± 0.8 times higher than the diffuse signal from the surrounding area) (Figure 6). The finger‐like structures which were discernible as filling defects in the latter subgroup of cells looked like metaphase chromosomes (Figure 6), suggesting that this subgroup was comprised of dividing cells. In these cells, the granular Barr2‐GFP structures seemed to be in close proximity to the chromosomes. There was no such accumulation in Barr1‐HEK cells (the dividing cell seen in the right‐center of Figure 3B).

**FIGURE 6:**
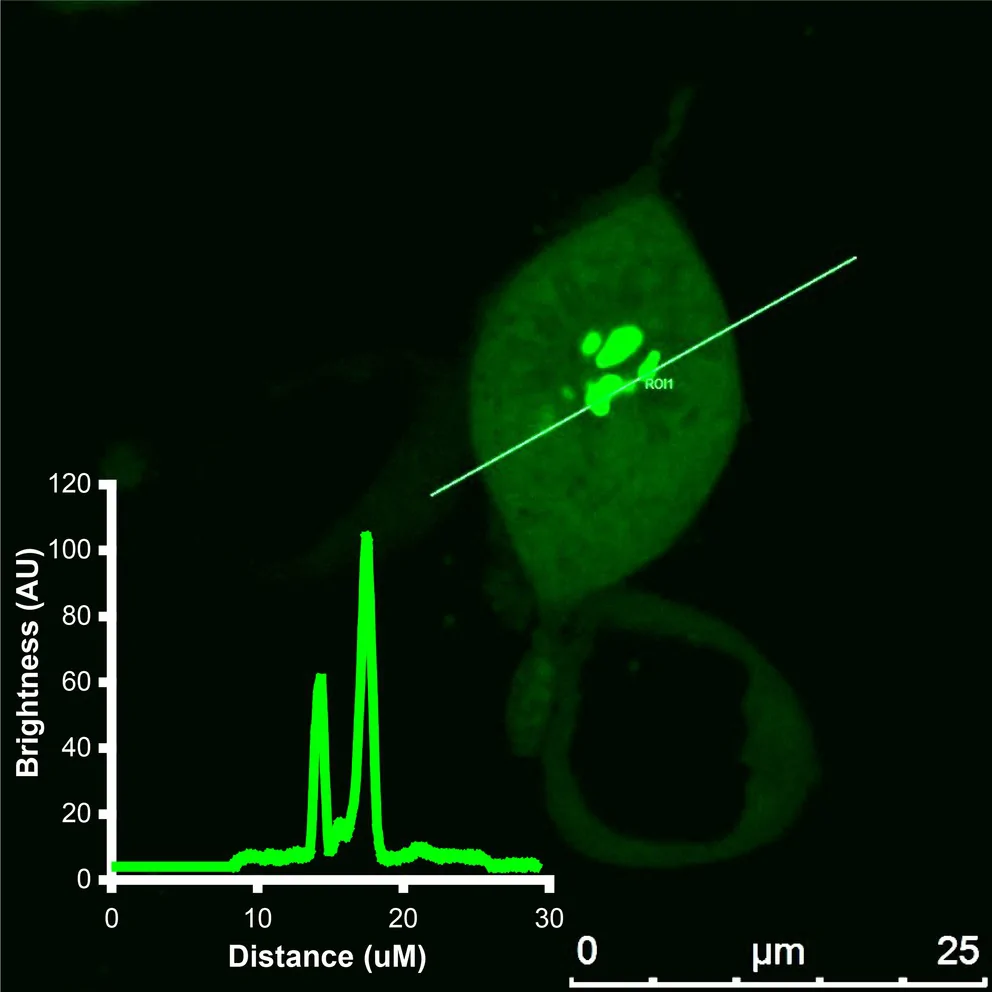
Barr2‐GFP accumulates in granular structures within a sub‐group of HEK‐293 cells. Live cells expressing Barr2‐GFP were imaged. Few Barr2‐GFP‐HEK cells (represented by a single cell in the image) do not have a discernable nucleus and show a homogenous Barr2‐GFP distribution within the cell except for the dark filling defects. Line‐scan data along the line drawn through cell (green line) is shown as an inset. Image shows a single optical slice. Drawn line is 30 μM long. Note that Barr2‐GFP signal can reach levels several folds higher within the granular structures compared to the signal in the cytoplasm. Similar cells can also be seen in Figures 4A, 7A, 8 and S2B, also for Barr1‐GFP in Figures 3B and 7B (however for Barr1‐GFP without any granular accumulation).

In order to test the assumption that Barr2‐GFP accumulates in spots associated with the chromosomes in dividing cells, we stained fixed Barr2‐HEK cells with the DNA dye Sytox Orange. Cells which showed a homogeneous Barr2‐GFP distribution with no visible nucleus actually had metaphase (or prometaphase) chromosome‐like structures that were stained with the DNA dye (Figure 7A,B), indicating that this subgroup was indeed comprised of dividing cells as we initially suspected. Confocal microscope analysis of the double‐labeled preparations showed that the granular structures with bright Barr2‐GFP signal observed in these cells were actually in close spatial association with the chromosome‐like structures (Figures 7A and S4, Movie S3). There was no visible accumulation of GFP signal in dividing Barr1‐GFP‐transfected cells (Figure 7B).

**FIGURE 7:**
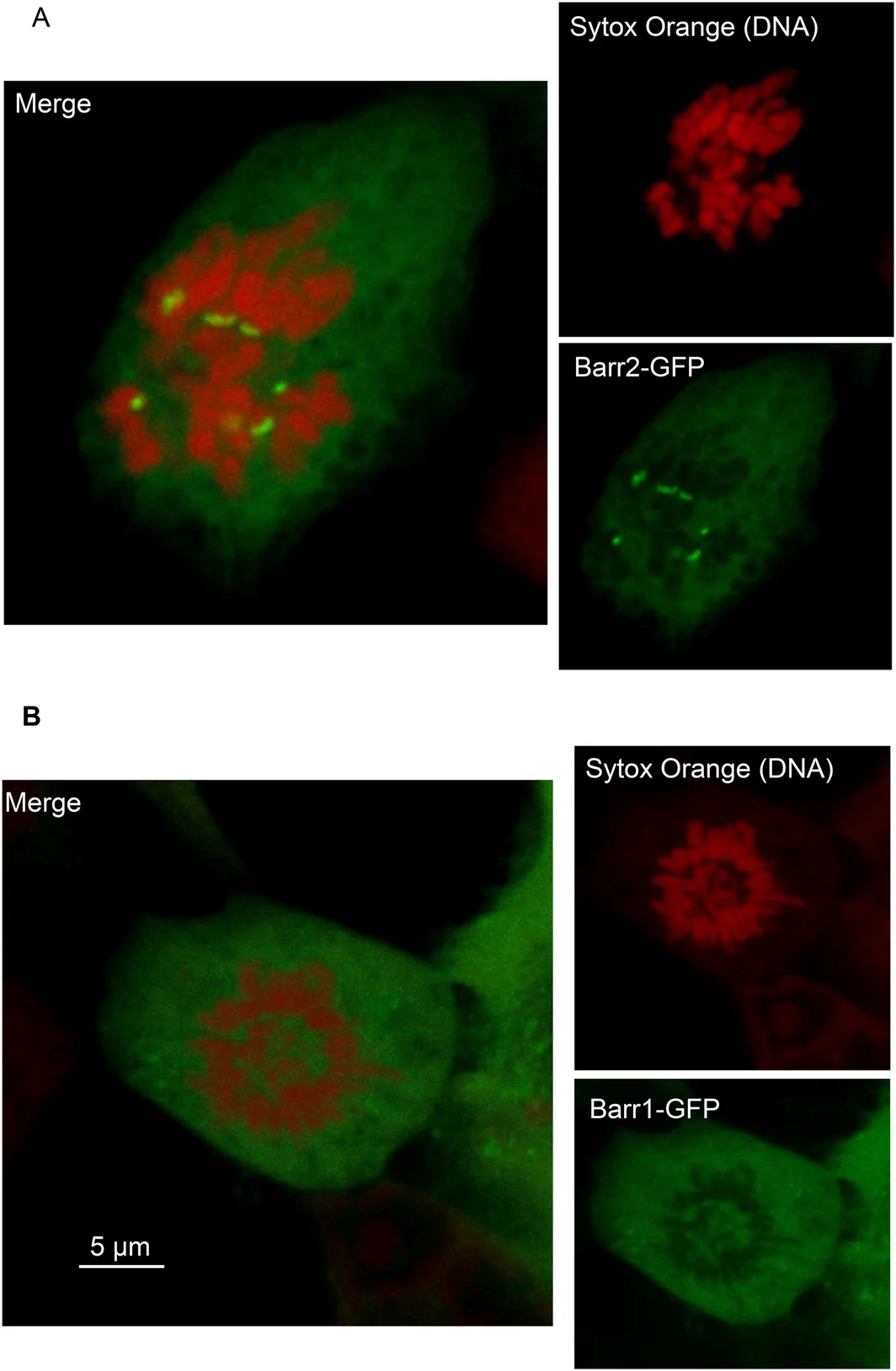
Granular structures containing Barr2‐GFP are associated with chromosomes in dividing HEK‐293 cells. Cells expressing Barr2‐GFP were fixed and stained with Sytox Orange to visualize chromatin and chromosomes. Images of Sytox Orange staining (red, right upper panels) and Barr2‐GFP signal (green, right lower panels), as well as a merged image (left panels) are presented. (A) The image shows a Barr2‐GFP expressing cell that is undergoing division with its chromosomes (red) visible. Note that the granular Barr2‐GFP (green) accumulations are closely associated with the chromosomes. (B) The image shows a dividing cell expressing Barr1‐GFP, where chromosomes (red) can also be seen, but no visible granular Barr1‐GFP (green) accumulation is present. Pictures represent 37 dividing cells from 5 independent experiments and 15 dividing cells from 4 independent experiments for Barr1‐GFP and Barr2‐GFP respectively. All the dividing cells we observed showed the same pattern without exception. Images show single optical slices. Scale is the same for all images and indicated on the figure. See also Figure S4 and Movie S3.

### 3.6 Barr2‐GFP Signal Colocalizes Only With UBF, but Not With Fibrillarin or Nucleolin in Dividing Cells

In contrast to the nondividing cells, no significant Fibrillarin or Nucleolin staining was observable in dividing cells (Figure S5A,B). However, a granular staining, which was as strong as the one seen in nondividing cells, was obtained with anti‐UBF antibody in the same dividing cells (Figure 8). This observation is in good agreement with the fact that UBF stays attached to the sections of the DNA that are an integral part of the nucleolus, whereas Fibrillarin and Nucleolin disperse as nucleoli disintegrate during cell division. Thus, unlike Fibrillarin or Nucleolin, UBF stays attached to the chromosomes throughout cell division and, when stained, is observed as small granular structures [24]. Importantly, Barr2‐GFP clumps that we observed in dividing cells showed almost perfect colocalization with the UBF staining within the optical resolution of the microscope (Figure 8, Movie S4). All the cells that were identified as dividing showed the staining and colocalization patterns described above, without any exception. Thus, apparently, in all dividing cells Barr2‐GFP accumulates to structures closely associated with metaphase chromosomes in close proximity with UBF. DNA sections on the chromosomes to which UBF protein strongly binds are known to be Nucleolar Organizing Regions (NORs). They form an important component of the nucleolus [16]. Hence, our present observations suggest that, during cell division, Barr2‐GFP binds to NORs (or to structures that are closely associated with them) on the chromosomes. All the dividing cells (that we could identify) showed the granular Barr2‐GFP accumulation, staining and colocalization patterns described in this and previous paragraph, without any exception.

**FIGURE 8:**
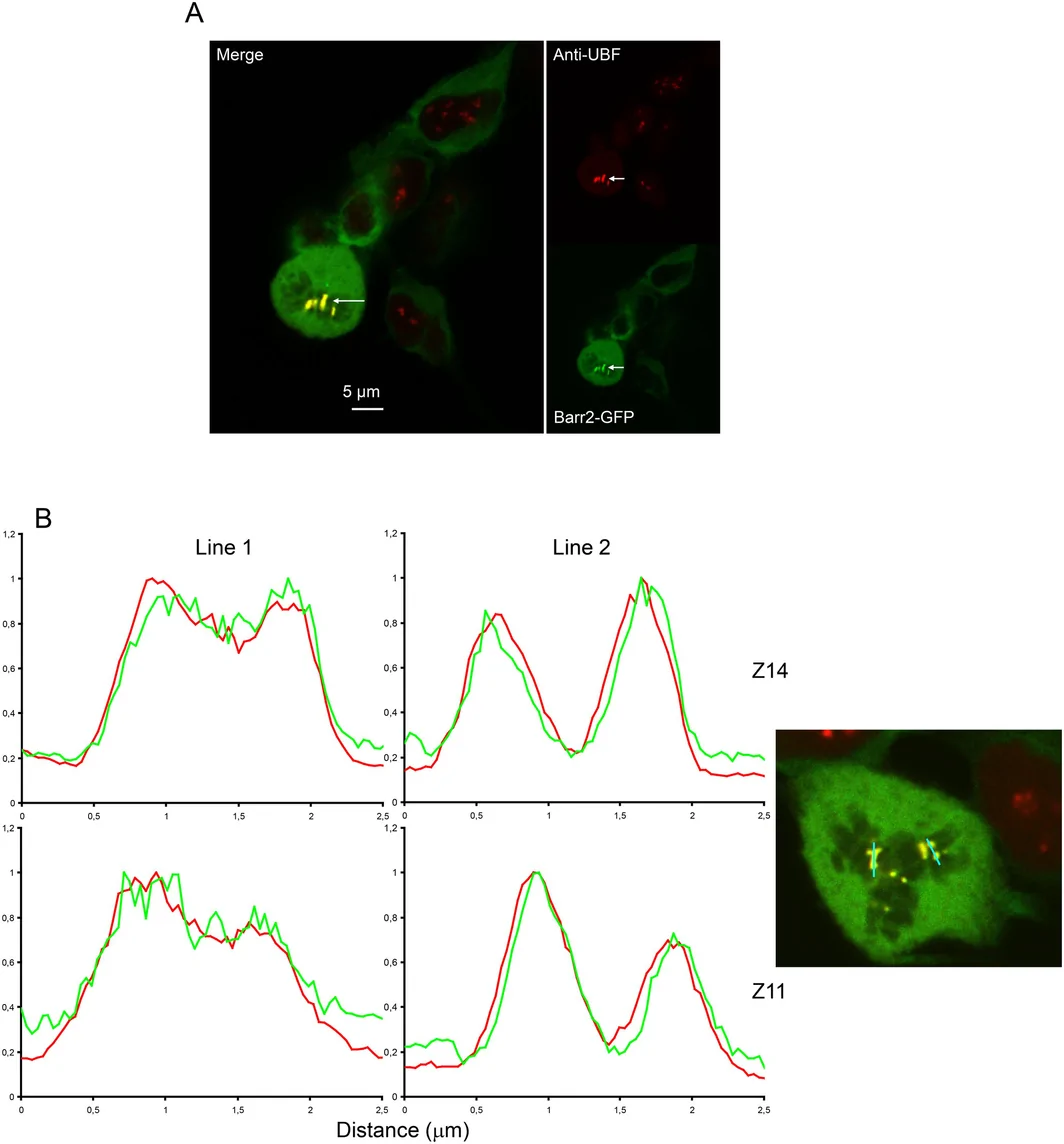
Chromosome‐associated granular Barr2‐GFP accumulations are colocalized with UBF. Cells expressing Barr2‐GFP were fixed and stained with anti‐UBF Ab. (A) Images of anti‐UBF staining (red) and Barr2‐GFP (green, right panels), as well as a merged image (left panel), are presented. Cell marked with the arrow is apparently undergoing division as it has no visible nucleus and metaphase chromosomes are discernable as filling defects in the diffuse cytoplasmic background formed by Barr2‐GFP. The arrow points to a group of granules that show both a bright Barr2‐GFP signal and a strong anti‐UBF staining. Notice that all Barr2‐GFP granules colocalize with anti‐UBF staining (For the lack of any anti‐Nucleolin or –Fibrillarin staining in the dividing cells see Figure S5A,B). Images show a single optical slice. Scale is indicated on the merged image. Image represents 38 cells from 10 independent experiments. All the dividing cells we observed showed the same pattern without exception. (B) For a different cell a single z‐slice of a merged image from Anti‐UBF Ab staining (red) and Barr2‐GFP signal (green) is presented on the right. On the left, line scan data for the two blue lines marked on the image are shown for two z‐slices that are 0.43 μm apart in depth (Z11 and Z14). Color‐coding is the same with the confocal image. Notice that distribution and localization of Barr2‐GFP and Anti UBF signal are almost identical in all z‐slices. Data are normalized to the maximum value of each line scan. Blue lines are 2.5 μm long. For a 3D reconstruction showing the colocalization between Barr2‐GFP and UBF in a different dividing cell see Movie S4.

### 3.7 Granular Accumulation of Barr2‐GFP During Cell Division Is Phase‐Dependent

Altogether, observations presented above suggest that Barr2‐GFP is transiently accumulated in the nucleoli in a specific phase during the cell cycle. In order to follow the whole process of this in‐and‐out of nucleolus trafficking and to support it further, we conducted experiments lasting between 180 and 210 min with Barr2‐GFP‐expressing living cells. As the start of cell division is an unpredictable random process and due to the time restrictions in our experiments, it was impossible to observe cell division from start to finish in the same cell. Thus, we stitched together two groups of experiments to observe the whole process; one includes cells at the initial phases of division (roughly from the disappearance of nuclear membrane to metaphase) and the second at a later phase (from metaphase onwards). Figure 9A,B represent such two experiments which together show the whole process: when a cell starts to divide, the nucleus which is clearly discernible due to its low Barr2‐GFP signal disappears and Barr2‐GFP shows a more homogenous distribution within the cell. This relatively fast event is most likely due to the breaking down of the nuclear membrane at the early stage of the cell division (prometaphase). Simultaneously, bright granular accumulation of the GFP signal within the cell becomes visible (Figure 9A, Movie S5). In telophase, after the separation of the daughter cells is completed, newly formed nuclei can be distinguished as the Barr2‐GFP concentration gradually decreases inside the reformed nuclear membrane. Bright Barr2‐GFP granules can be seen within the nucleus for a while. However, as the exclusion of Barr2‐GFP from the nucleus continues, these aggregates also gradually dim out and eventually disappear within a few hours after the division is completed (Figure 9B, Movie S6). Movies S5 and S6 show the whole sequence of images. Different dividing cells can also be seen in Movie S7.

**FIGURE 9:**
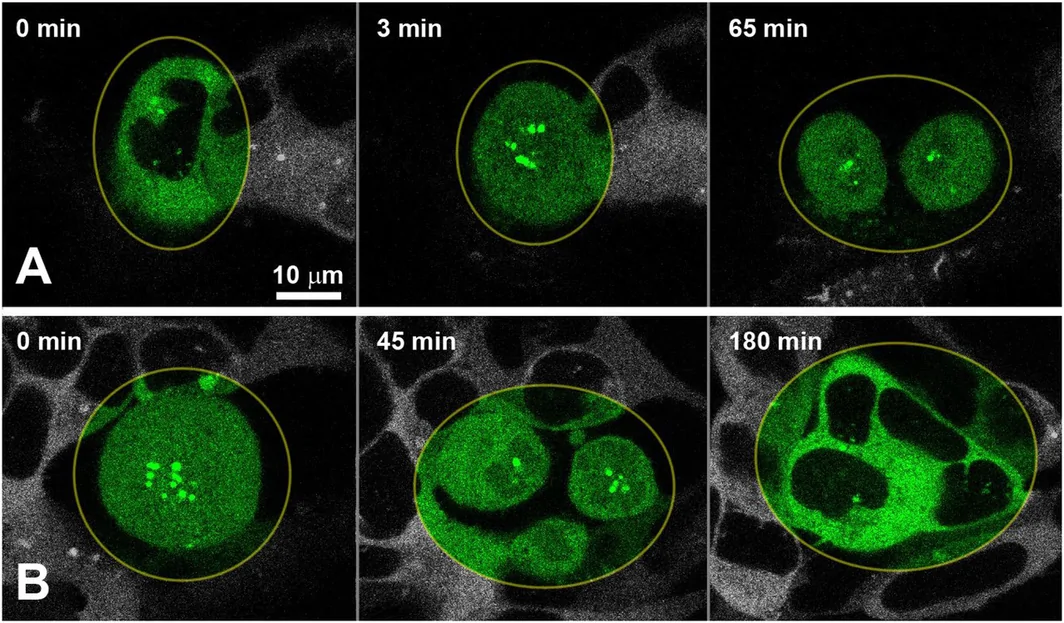
Redistribution of Barr2‐GFP between cytoplasm and the granular structures during cell cycle. Consecutive z‐stack images of live Barr2‐GFP expressing cells were obtained every 3 min for 3 h. The image sequence in (A) starts with a cell at an early phase of cell division where nuclear membrane is still intact. The sequence in (B) starts after the disintegration of the nuclear membrane. Time in minutes indicate the time after the nuclear membrane starts to disappear in (A) and the time after the cell starts to split in (B). The tracked cell and its daughter cells are encircled in all images. (A) Note that granular spots of Barr2‐GFP accumulation are already formed at 3 min. (this image is the first image taken after the nuclear contours are lost) and stay visible at 65 min. when the cell division is completed. (B) Series of images shows a different cell starting from a later stage when clear granular Barr2‐GFP accumulations have already formed. At 45 min, after the cell division, the nuclear Barr2‐GFP concentration begins to decrease (indicating the reformation of nuclear membranes), making the nuclei discernable. Granular Barr2‐GFP accumulation is still prominent at this point. At 180 min, nuclei are now clearly visible and granular Barr2‐GFP accumulations have faded considerably. (Please refer to the Movies S5 and S6 which show the entire sequence of images and also Movie S7 of a different cell that undergoes cell division). Images show single confocal slices from selected time points. Scale is the same for all images and indicated on the figure. Images represent 7 independent long‐term experiments.

We also observed and imaged the Barr2‐GFP expressing cells for 5 h in an environmentally controlled microscopy system. We performed 5 independent experiments and observed 145 cell division events in total. All of those events showed initially the disappearance of the cell nucleus contours and a homogeneous distribution of Barr2‐GFP with distinct Barr2‐GFP puncta. In accordance with the results explained above, Barr2‐GFP puncta got distributed between the daughter cells after the cell separation. Eventually, reformation of the nuclear contours containing the intranuclear puncta was observed. In cases where the whole process could be traced, the intranuclear puncta disappeared within 2–3 h. An exemplary division event and a stitched field of view for one of the experiments can be seen in Movie S8.

These results clearly show that nucleolar Barr2‐GFP accumulation occurs not only with P2X7R stimulation but also in non‐stimulated cells during cell division and continues for a limited time after cell division is completed. They also highlight the importance of tracking the cells during P2X7R stimulation and visually identifying the ones responding to the ATP application in order to distinguish them from the cells which already show a granular Barr2‐GFP accumulation prior to stimulation. Furthermore, they show that it is important to distinguish these ATP‐responding cells from cells going into cell division. DNA staining of the live cells that are tracked and identified as responders to P2X7R stimulation clearly shows that these ATP‐responding cells do not have any metaphase chromosomes but only loose chromatin, indicating that they are nondividing cells (Figure 2B). This further strengthens our conclusion that the Barr2‐GFP accumulation after ATP application was indeed due to P2X7R stimulation.

## 4 Discussion

We observed that Barr2‐GFP could accumulate in granular structures within the nucleus during two distinct and unrelated events: (i) P2X7R stimulation and (ii) cell division. In both events, the granular accumulation appears to be within the nucleoli. In the case of the dividing cells, accumulation seems to occur on certain regions of the metaphase chromosomes. The granular Barr2‐GFP signal induced by P2X7R in nondividing cells displays a strong association with nucleolar proteins Fibrillarin and Nucleolin, and especially with UBF. In dividing cells, the Barr2‐GFP signal is strongly associated only with the UBF protein but not with Fibrillarin or Nucleolin. As UBF is known to strongly bind to NORs [16, 24], we concluded that these granular accumulations, whether within the nucleoli or on the condensed chromosomes, occur on NORs (or a closely associated structure). In nondividing cells following the CRM‐1 pump inhibition by LMB, the intranuclear concentration of Barr2‐GFP increases and Barr2‐GFP accumulates into granular structures which also show strong association with nucleolar proteins. In both P2X7R stimulation and cell division, the nucleolar accumulation seems to be quite specific to Barr2. Even though its intranuclear concentration can also be increased by P2X7R stimulation, Barr1‐GFP, a closely related protein to Barr2, does not show any such accumulation.

Present results showing that compromising the nuclear membrane barrier either by LMB application or possibly by P2X7R stimulation increases intranuclear Barr2 concentration and leads to its nucleolar accumulation are in good agreement with published results using a NES mutated Barr2. This mutant cannot interact with the CRM‐1 pump and thus has an elevated nucleoplasmic concentration and can also readily accumulate into the nucleoli [6]. Hence, it can safely be concluded that increasing the nucleoplasmic concentration of Barr2‐GFP is sufficient to trigger its accumulation within the nucleoli. Our observations showing that Barr2 is in close proximity with nucleolus specific proteins Nucleolin, Fibrillarin and UBF is also in agreement with results of the same study where the NES‐compromised Barr2 was found to interact with several important nucleolar proteins involved in the biosynthesis of ribonucleoprotein complexes and RNA splicing, including UBF and Fibrillarin [6]. The results of a proteomics study have also suggested that Beta‐arrestins may interact with nucleolar proteins [7]. Taken together, these findings strongly suggest that Barr2, provided that it is not excluded from the nucleus one way or the other, binds and interacts with proteins associated with nucleoli or NORs. Moreover, this interaction seems to be specific for Barr2‐GFP, as Barr1‐GFP does not display any such interaction. In other words, Barr2, but not Barr1, has an intrinsic affinity for some nucleolar proteins. Clearly, the contact between Barr2 and the nucleolar proteins is prevented by the action of the CRM‐1 pump.

Another important finding of the present study is that Barr2‐GFP accumulates into NORs and then into the nucleoli during cell division. Real‐time observations in living cells showed that Barr2‐GFP accumulated into granular structures during cell division right after the disappearance of nuclear membranes. These accumulation spots formed without any receptor stimulation and were closely associated with the metaphase or anaphase chromosomes of the dividing cells. Double staining in dividing cells revealed that Barr2‐GFP almost exactly colocalized with the UBF protein on chromosomes. UBF is a protein known to strongly bind to the structures called NORs [16, 24]. Hence, at the onset of cell division, with the disappearance of the nuclear membrane and thus the elimination of this diffusion barrier, Barr2‐GFP seems to reach the chromosomes and the NORs. Most probably (as suggested by the findings discussed above) due to its intrinsic affinity to a NOR‐associated component(s), Barr2‐GFP binds to the NORs and stays bound all through the cell division (Figure 10B Stages II and III).

**FIGURE 10:**
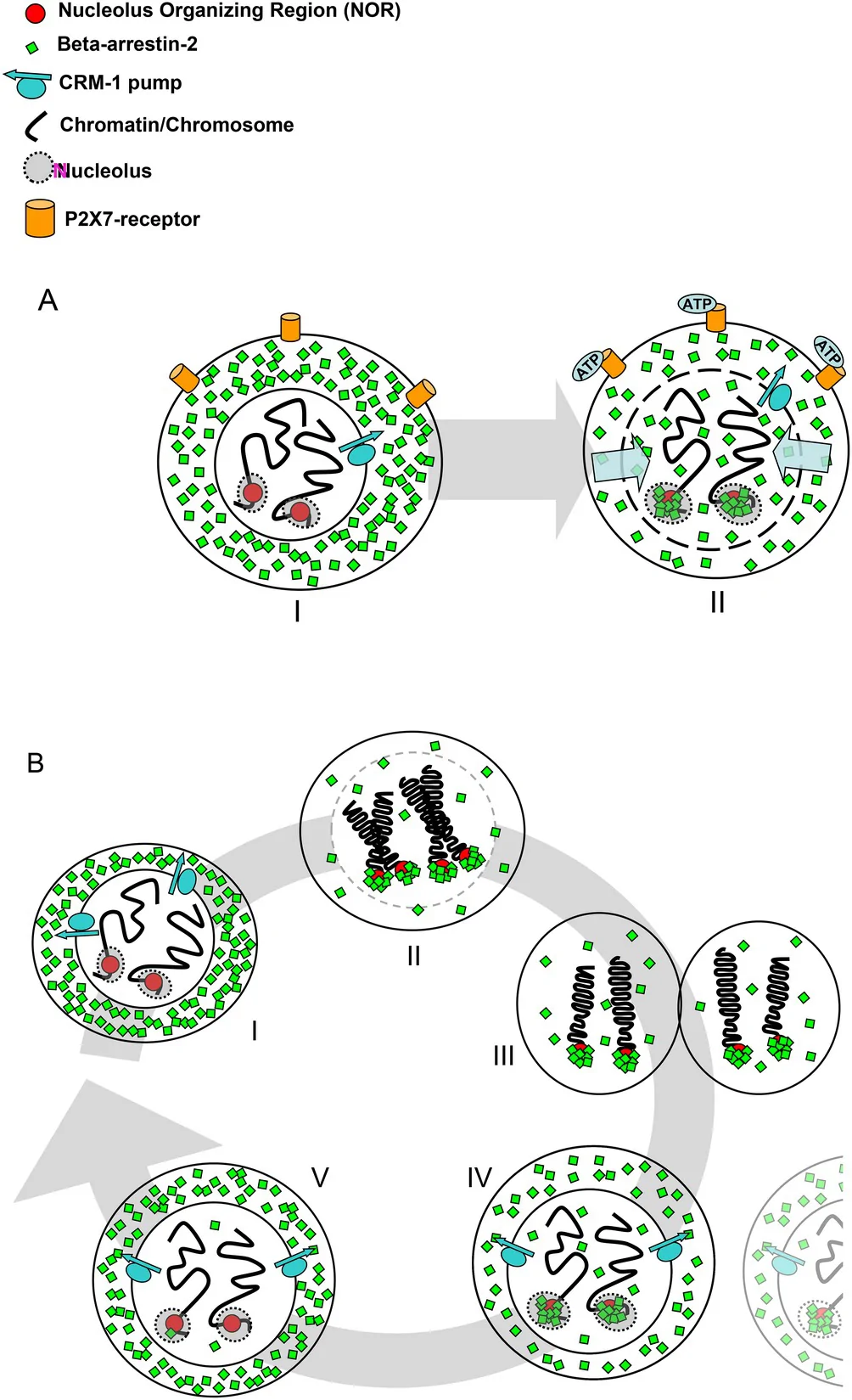
Two modes of granular Barr2‐GFP accumulation in HEK‐293 cells. In the resting cell, Barr‐2 is mainly a cytoplasmic protein with a very low concentration within the nucleus due to CRM‐1 pump activity (I. in both panels A and B). (A) P2X7 receptor stimulation‐induced aggregation into the nucleoli; P2X7 receptor stimulation (II) compromises the nuclear membrane barrier, and Barr2‐GFP now can enter into nucleoplasm and accumulate into the nucleoli. (B) Cycling behavior of Barr‐2 between NORs and cytoplasm during the cell cycle; in a resting cell (I) with the initiation of cell division (II), the nuclear membrane disintegrates and, due to elimination of this barrier, Barr‐2 can now access and bind to the NORs on the condensed chromosomes. After the division is completed (III), Barr‐2 stays attached to the NORs until the reformation of the nuclear membrane in the daughter cells. With the reformation of the nuclear membrane (IV), CRM‐1 pump now can carry Barr‐2 out of the nuclear space into the cytoplasm, decreasing its concentration within the nucleus. During the early stages of this phase, Barr‐2 stays attached to the NORs, and thus to the newly formed nucleoli, until the Barr‐2 concentration in the nuclear space drops significantly. Eventually (V), Barr‐2 concentration decreases to very low levels within the nuclear space, and the bound Barr‐2 dissociates from the NORs (and thus from the nucleoli).

NORs are stretches of DNA on the acrocentric chromosomes that contain rRNA‐coding DNA repeats. They also form the core of the nucleoli during interphase [16]. Nucleoli are known to disintegrate during prophase and proteins forming the nucleolus such as Fibrillarin disperse as the condensed chromosomes are formed [16, 25]. However, UBF is known to stay associated with NORs all through the cell cycle. Thus, UBF appears both as a nucleolus‐associated protein during interphase and also as a chromosome‐associated protein during metaphase and anaphase [16, 24]. In good agreement with this fact, we observed that in dividing cells, while granular staining with anti‐Fibrillarin or anti‐Nucleolin antibodies disappeared, anti‐UBF antibody showed a granular staining associated with the chromosomes. Real‐time observations also suggested that following metaphase, after the reformation of the new nuclear membranes (and also the reformation of the new nucleoli) in the daughter cells, Barr2‐GFP continued to be associated with NORs and hence with the newly forming nucleoli (or probably with prenucleolar bodies; PNBs). However, this association of Barr2‐GFP with the nucleoli continued only for a limited time; with the removal from the nucleus into the cytoplasm (presumably by the NES‐pump complex) and the drop of its nuclear concentration, Barr2‐GFP dissociated from these sites in the nucleoli. Thus the intra‐nucleolar Barr2‐GFP accumulation could only be seen in recently divided cells (Figure 10B Stages IV and V). This explains why only a small fraction of the interphase cells show a nucleolar Barr2‐GFP accumulation. Apparently, Barr2‐GFP stays at close association with NORs during most of the mitotic phase and late telophase, as it seems to be aggregated in granular structures which strongly colocalize with UBF during this whole period. This close association both on the metaphase chromosomes of the dividing cells and also in the nucleoli of the cells in telophase strongly suggests that the binding target of the Barr2‐GFP may either be UBF itself, or NORs themselves (or a very closely associated region on the chromosomes).

Based on the optical resemblance as well as colocalization similarities between the granular Barr2‐GFP accumulations observed during cell division and those that are induced by P2X7R stimulation and given the apparent intrinsic affinity of Barr2‐GFP for the nucleoli and NORs, we deduce that the binding target of Barr2‐GFP following P2X7R stimulation is also a region on the chromosomes close to NORs. A similar Barr2 accumulation to what we observed after P2X7R stimulation was also observed with TRPV1 receptor stimulation [5, 6]. That accumulation was suggested to play a role in TRPV1 signal transduction. As mentioned above, TRPV1 and P2X7 receptors share interesting properties; they are both ligand‐gated cation channels, they both allow the passage of large molecules through the cell membrane, and they both cause extensive blebbing and cell shape changes [26, 27]. Thus, it may be proposed that Barr2 translocation may be a general and novel signal transduction mechanism for a set of receptors that are currently thought to function only as ligand‐gated ion channels. However, one should take into consideration that in both cases, TRPV1 receptor stimulation and our observations with the P2X7R stimulation, only a fraction of the cells shows the Barr2 accumulation response. Additionally, the overexpression of both the TRPV1 and P2X7R is known to induce large ionic currents and extensive changes in cell morphology and may lead to cell death or trigger programmed cell death. Even though our experiments with P2X7R stimulation were performed in bath solutions to minimize intracellular ion concentration changes (no added Ca^++^ and high K^+^ bath solutions), it must be taken into consideration that rather than being a signal transduction mechanism, nucleolar translocation of Barr2 may be the result of other processes such as cell death. At this point, our results strongly suggest that P2X7R induces the nucleolar accumulation of Barr2‐GFP by increasing its nucleoplasmic concentration (possibly along with other proteins excluded from the nucleoplasm), and not through a specific interference with the CRM‐1 pump function, but probably through a more general compromise of the nuclear barrier function (Figure 10A).

It is important to emphasize that the phenomenon we report here is different from the nucleolar accumulation of certain viral proteins described earlier (See the review by Hiscox; [28]). It does not resemble the sequestration of proteins into the so‐called “detention centers” either. This type of protein accumulation within the nucleoli by binding to long noncoding RNAs is seen after the exposure of the cells to heat shock or acidosis [29, 30]. Our observations indicate that the nucleolar Barr2‐GFP accumulation is a cyclical phenomenon occurring only for a limited time period during the normal cell cycle, specifically from prophase up to the late telophase, rather than a stress‐induced response or a steady‐state situation. Additionally, unlike the viral protein accumulation or stress‐induced sequestration, both of which were reported to concern the whole nucleolus, the accumulation of Barr2‐GFP appears to be related to the NORs. The Barr2‐GFP accumulation we report here is also clearly different from Barr2 accumulation in centrosomes that was reported previously [8]. Perinuclear spots of Barr2‐GFP fluorescence that are visible in some optical slices may well represent this centrosomal accumulation. However, we did not explore these accumulations further.

Clearly, functional consequences of Barr2 accumulation onto NORs require further research. However, a short discussion on the issue can be instructive for further studies. A recent study demonstrated that Barr1 and Barr2 can show a phenomenon called “Liquid Liquid Phase Separation (LLPS)” and it was suggested that this property is crucial for the functioning of Barr1 and Barr2 [31]. Many signaling molecules are known to function through forming condensates/droplets within the cytoplasm. LLPS is understood to be an important mechanism for the formation of these condensates [32]. This condensate formation, in turn, seems to play an important role in the accumulation of reactants and the compartmentalisation of chemical processes within the cell, without the need for physical barriers such as lipidic membranes [33, 34]. The Barr condensates reported by Anderson et al. formed cytoplasmic puncta that are similar to the puncta we observed near the NORs. The authors also showed that the Barr1 and 2 condensates are regulated by the GPCR activation [31]. This opens up the intriguing possibility that the Barr2‐GFP accumulation that we observed can be a Liquid Liquid Phase Separation induced by the interaction between Barr2 and some entity near or on the NORs.

We may define two main classes of possible functional role for the observed Barr2 accumulation: (1) Functions that may occur at the site of the protein accumulation; (2) Functions that may be due to the sequestration of Barr2 at NORs, thus preventing other probable interactions of the protein elsewhere in the cell during the mitotic phase. For the second group of functions, one clue may be the finding in [31] indicating that GPCR activation may decrease the cytoplasmic Barr condensates, which may be interpreted as condensates containing the inactive form. As for the first group of functions, it has been suggested that, after TRPV1 receptor stimulation, Barr2 accumulates into the nucleolus, interacting with RNA polymerase I and thus increasing its activity and decreasing the levels of tumor suppressor p53 [6]. As another possible action, one may consider a modulatory effect on the disassembly‐reassembly of the nucleoli during and after cell division by the binding of Barr2 to NORs. Nucleolar assembly‐disassembly is a process controlled and modulated by many players. Interestingly LMB, a CRM‐1 pump complex (NES‐pump) inhibitor, has been reported to impede the reformation of the nucleoli after the mitotic phase by arresting them at a certain stage of assembly [23]. This effect was attributed by the authors to a not‐yet‐determined action of LMB which is unrelated to inhibition of CRM‐1 pump function (see [35] for a review of those nontransport functions). However, it can easily be assumed that after cell division CRM‐1 pump may remove a hypothetical nucleoli‐dissociating factor(s) from the newly formed nucleus, and as a result, it allows the reformation of the nucleoli. In that case, LMB‐induced arrest of nucleolar reassembly can be attributed to the CRM‐1 pump inhibition and prevention of the removal of this hypothetical factor. Such a nucleoli‐dissociating factor has already been hypothesized by Leung et al. [24] to explain the dissociation of the nucleoli during cell division. According to the authors, this factor should normally be excluded from the nucleus but should also be able to gain access to the nucleoli during the cell division upon disintegration of the nuclear membrane. This way, it may induce dissociation of Fibrillarin from the nucleoli. As explained in detail above, during the cell division Barr2 seems to transiently associate with NORs, which are central for nucleolus formation. After the cell division is completed, Barr2 is transported out of the nucleus by the CRM‐1 pump in concert with the reformation of the nucleoli. These properties make Barr2 a good candidate for such a nucleoli‐dissociating factor (or one of several interacting factors). Additionally, the previously reported, constitutive binding and regulation of centrosome function through Barr2 [8], taken together with our results, may indicate a much more widespread role of Barr2 protein in cell division.

Our observations additionally suggested the presence of at least two different nuclear export pumps; one for Barr2 and the other for Barr1. It can be seen in Figure 5A–C that when the difference between the expression levels is compensated, both Barr1‐GFP and Barr2‐GFP proteins are excluded from the nucleus to a similar extent. Even though LMB treatment dramatically changes the nucleoplasmic to cytoplasmic signal ratio of Barr2‐GFP, it has absolutely no effect on Barr1‐GFP distribution (Figure 5C). This suggests the presence of two distinct nuclear export pumps; one for Barr2 export which is sensitive to LMB inhibition, and a second one for Barr1 which is not. Our finding that both Barr1 and Barr2 are effectively excluded from the nucleus is in agreement with some of the previous reports [10, 21] especially with Song et al.'s [22]. However, it differs from Scott et al. [9] and Wang et al. [11], as they observed no exclusion from the nucleus for Barr1 in the basal conditions. This discrepancy may suggest that the expression or function of nuclear export pumps may change depending on the cell type or even on the state of the same cell. This point gains importance as our study suggests that the exclusion of Barr2 from the nucleoplasm by a nuclear export pump appears to be the main mechanism that regulates the interaction between Barr2 and NORs/nucleoli.

Our present findings suggest that Barr2, a molecule known for its important and diverse roles in GPCR signal transduction, may have completely different NOR‐related novel roles in cell physiology, especially in cell division. Further investigation of the phenomenon reported here will illuminate what such physiological roles are.

## Author Contributions

S. Uysal and M. Uğur designed the research; T. Gümüştaş, I. Casella, S. Uysal, M. Uğur, and Ö. Uğur performed the experiments; T. Gümüştaş, S. Uysal, and M. Uğur analyzed the data; M. Uğur wrote the paper.

## Funding

This work is supported by the Scientific and Technological Research Council of Turkey (TUBITAK) (grant 113S376).

## Conflicts of Interest

The authors declare no conflicts of interest.

## Supporting information


**Figure S1:** Barr1‐GFP does not accumulate into granules within the nuclei, on the contrary excluded from small structures resembling nucleoli. Live cells were incubated with vehicle (A) or LMB (B) and imaged as described in Figure 1. Lower row of images are obtained from the same area shown in the upper row, using a higher gain (higher laser power and PMT gain, as a result of this the bright parts of the images saturate). Compared to the vehicle treatment (A, left column, upper row, labeled as “Control”) overnight LMB treatment (B, left column, upper row, labeled as “LMB +”) does not cause any granular Barr1‐GFP accumulation. Nucleoplasmic concentration of Barr1‐GFP does not increase either. However, in Barr2‐GFP cells, compared to control (A, right column, upper row, labeled as “Control”) overnight LMB treatment (B, right column, upper row, labeled as “LMB +”) significantly increases the number cells with bright granular Barr2‐GFP accumulation within the nuclei and also the brightness of the granules increases. LMB treatment also increases the diffuse nucleoplasmic concentration of Barr2‐GFP. No effect of LMB on the granular aggregation or nucleoplasmic concentration of Barr1‐GFP is visible in the higher‐gain images of the same areas either (compare Panel A and B; left columns, lower row). In these higher‐gain images where some diffuse nucleoplasmic Barr1‐GFP signal is observable, Barr1‐GFP is seen to be clearly excluded from small areas within the nucleus (red arrows) resembling nucleoli. This is in stark contrast to Barr2‐GFP where high gain images further reveal the presence of granular Barr2‐GFP aggregation (green arrows) in the nucleus in some control cells (A, right column, lower row). Also no diffuse nucleoplasmic Barr2‐GFP signal is observable in the high gain images of the control cells.

**Figure S2:** LMB‐induced granular Barr2‐GFP accumulation is also located to the nucleolus. Barr2‐GFP expressing cells were treated with LMB and stained with anti‐Fibrillarin (A), anti‐Nucleolin (B) or anti‐UBF (C) Abs. Separate images of Ab staining (red) and Barr2‐GFP (green, right panels), as well as a merged image, are presented (left panel). LMB treatment causes Barr2‐GFP accumulation which is in close spatial association with nucleolar proteins (white arrows). Red Arrows indicate exemplary structures unique to LMB‐treated cells (features not observed in untreated cells). First type of structure is the “pairs” formed between Barr2‐GFP and anti‐Fibrillarin and also anti‐UBF staining. In those “pairs” even though the Barr2‐GFP and Ab signals are in very close proximity, two very distinct round areas in contact with each other can be discerned (See also Figure S3). Second type of structure shows Barr2‐GFP accumulation without any nucleolar protein staining. Note that, except for these differences, the staining patterns of Fibrillarin, Nucleolin and UBF in LMB‐treated cells are similar to their counterparts in untreated cells. All cells observed to have granular Barr‐2 accumlation showed this behavior without exception.

**Figure S3:** “Pairing” behavior between Fibrillarin and Barr2‐GFP seen in LMB‐treated cells. Cells expressing Barr2‐GFP were treated with LMB and stained with anti‐Fibrillarin Ab. At the top, separate images from red (Anti‐Fibrillarin Ab staining) and green (Barr2‐GFP) channels are displayed. At the bottom a merged image is presented. Image is from a different cell but it is similar to the one in Figure S2, only in higher magnification. Despite the close spatial association between anti‐Fibrillarin staining and Barr2‐GFP, similar to non LMB treated cells, Barr2‐GFP and anti‐Fibrillarin Ab staining show different distributions on the sub‐micron scale (white arrows). Red Arrows indicate “pairing” behavior between Barr2‐GFP and anti‐Fibrillarin staining similar to the one seen in Figure S2. This “pairing” behavior is a peculiarity of LMB treatment and not observed in untreated cells.

**Figure S4:** Barr2‐GFP Accumulates into Granular Structures Closely Associated with Metaphase Chromosomes. An untreated dividing cell which expresses Barr2‐GFP is shown. Fixed cells were stained with Sytox Orange to reveal the chromosomes. Separate images of Sytox Orange staining (red) and Barr2‐GFP (green) signal, and also a merged image, are shown. The cell shown at the center of the image is at anaphase stage and Barr2‐GFP can be seen to form granular structures in close proximity with chromatids at each pole.

**Figure S5:** Neither Fibrillarin nor Nucleolin Display any Granular Structure in Dividing Cells. Barr2‐GFP expressing untreated dividing cells are shown. Fixed cells were stained with anti‐Fibrillarin (A), and anti‐Nucleolin (B) Abs. Separate images of Ab staining (red) and Barr2‐GFP (green), as well as a merged image, are presented. Neither the anti‐Fibrillarin nor the anti‐Nucleolin Ab stains a visible structure inside the dividing cell (compare the staining in the surrounding nondividing cells). In contrast, Barr2‐GFP shows clear granular accumulation (arrows). Compare this result with Figure 8 where anti‐UBF Ab clearly stains granular structures that are closely associated with Barr2‐GFP in a dividing cell.

**Movie S1:** P2X7 receptor stimulation causes granular accumulation of Barr2‐GFP. P2X7 receptor and Barr2‐GFP expressing live HEK‐293 cells are shown. The video is constructed by merging z‐slice images. At around 5 min P2X7 receptor agonist ATP is added to the medium. ATP stimulation causes granular accumulation of Barr2‐GFP in some cells (some examples indicated by arrows). It can be seen that in these cells, nuclear Barr2‐GFP concentration also increases. Note that some cells already show granular Barr2‐GFP accumulation prior to ATP application (For example lower left corner) they should be distinguished from the newly formed granules following the P2X7 receptor stimulation. ATP causes blebbing and movement in the cells, a well known effect of P2X7 receptor stimulation. The counter at the lower left indicates experiment time as hours: minutes: seconds. milliseconds.
**Movie S2:** P2X7 receptor stimulation does not cause granular accumulation of Barr1‐GFP. P2X7 receptor and Barr1‐GFP expressing live HEK‐293 cells are shown. ATP is added to the medium at the beginning of the video. In the highlighted cells, ATP stimulation causes an increase in the nucleoplasmic concentration of Barr1‐GFP, but no granular accumulation is observable. Note that in order to clearly show the intra‐nuclear Barr1‐GFP increase video is constructed from single z‐slices, not merged images as in Movie S1. The counter at the lower right indicates experiment time as hours: minutes: seconds. milliseconds.
**Movie S3:** 3D image reconstruction of a dividing Barr2‐GFP‐HEK cell stained with Sytox Orange. Barr2‐GFP expressing HEK‐293 cell, fixed and stained with Sytox Orange to reveal the chromosomes. The video shows the 3‐D reconstruction of the z‐stack images of the dividing cell shown in Figure 7A to further illustrate the spatial association between the Barr2‐GFP and the chromosomes.
**Movie S4:** 3D image reconstruction of a dividing Barr2‐GFP‐HEK cell stained with anti‐UBF Ab. Barr2‐GFP expressing HEK‐293 cell fixed and stained with anti‐UBF Ab. The video shows the 3‐D reconstruction of the z‐stack images of a dividing cell. In order to highlight the colocalization of Barr2‐GFP and UBF, Barr2‐GFP signal colored green and UBF signal colored magenta thus their colocalization appears as white. Video illustrates the high correlation between the signal distributions of Barr2‐GFP and UBF all through the cell volume. This is a dfferent cell from those presented in Figure 8.
**Movie S5:** During cell division, with the disappearance of nuclear membrane Barr2‐GFP accumulates into granular structures. Barr2‐GFP expressing live, HEK‐293 cells are shown in the process of division. The cell can be seen to lose its nucleus and immediately afterwards, granular accumulation of Barr2‐GFP occurs. Then the cell proceeds to division. Note that this video does not show merged z‐stack images and as the cell lifts up from the bottom during the cell division, progressively higher z‐slices are used in order to keep the confocal‐plane in the middle of the tracked cell as much as possible (also see the explanation for Movie S7). This z‐stack shift can be noticed around 1:06:00 (number in the upper left counter). Upper left counter indicates experiment time as hours: minutes: seconds. milliseconds.
**Movie S6:** During cell division, after daughter cells separate Barr2‐GFP stays granular until the reformation of the nuclear membranes then the intensity of the granules slowly decrease. Barr2‐GFP expressing live, dividing HEK‐293 cells are shown. The video starts with a cell at a later stage of division than shown in Movie S5. Nuclear membrane has already disappeared and granules of Barr2‐GFP are already present. The cell proceeds to division. Nuclear membranes reform in the daughter cells. Barr2‐GFP concentration in the nuclei drops, nuclei become discernable, and Barr2‐GFP granules gradually fade. Upper left counter indicates experiment time as hours: minutes: seconds. milliseconds. Please note that Movie S5 and S6 were both prepared from a single experiment where two cells at different stages of division can be observed in the same field of view. Both videos first show the whole field of view and then the area of interest where the tracked cell and its daughter cells are highlighted. The video is composed of pictures from a single z‐slice, not from merged images. As two cells are at different heights (z‐positions) two videos show different z‐slices of the experiment. To follow the upward movement of the cells during the cell division, the z‐slice which the frame was taken was also moved up during the course of the video (also see Movie S7).
**Movie S7:** Another example of appearance and disappearance of granular Barr2‐GFP accumulation during cell division. Barr2‐GFP expressing live, dividing HEK‐293 cells are shown. This video shows a different experiment from the one in Movies S5 and S6. In those movies as the cells move in and out of the confocal plane the granular Barr2‐GFP accumulations tend to appear and disappear in single z‐plane images, making it difficult to judge the real brightness of the Barr2‐GFP aggregates. Thus, we composed this video by merging many of the z‐stack images in order to alleviate this artifact and allow a better assessment of the change in the brightness of the Barr2‐GFP aggregates. However this naturally makes it more difficult to differentiate the intra nuclear and cytoplasmic Bar2‐GFP signals. The video shows both a cell at an early, and another cell at a later stage of division. The cell at the early stage of cell division (left side of the frame, just below the middle line) loses its cell nucleus (around 2:27:00) and immediately following this event granular Barr2‐GFP aggregates appear within the cell. The second cell which is at a later stage of division (almost at the middle of the frame) had already lost its nucleus and the granular Barr2‐GFP aggregates had been formed when the recording started. It proceeds to cell split (around 00:42:00) and after the cell separation is completed, within the daughter cells nuclei reform. As Barr2‐GFP signal gradually decreases in the nuclei the granular Barr2‐GFP aggregates also lose their intensity. When both cells are taken together the appearance and disappearance of the granular Barr2‐GFP accumulation throughout the whole division process can be followed. Upper left counter indicates experiment time as hours: minutes: seconds. milliseconds.
**Movie S8:** Dividing cells observed in a 5% CO2, 37°C Environment Bathed in 10% Serum Containing DMEM. Cells were kept in an environment‐controlled fluorescent microscopy system at 37°C and 5% CO2, in DMEM for 5 h and images were taken every 15 or 30 min from multiple adjacent fields of view. 25 z‐stack images were recorded for each field of view using a 63X water immersion objective. In the video, initially a typical general view obtained with automatic stitching of all fields is shown. Then, a typical cell division is shown in close‐up. For the construction of the video, the cell was tracked through different z‐stacks, as it moves on the z‐axis during division. We performed 5 independent experiments and observed 145 cell division events. As can be seen in the video, in all of these division events at the beginning of the cell division, as soon as the nuclear membrane disintegrates accumulation of Barr2‐GFP into multiple puncta is observed. After cell separation occurs, the Barr2‐GFP puncta are separated to the daughter cells. With the formation of the nuclear membrane, Barr2‐GFP puncta stay in the nucleus for a while and eventually disappear approximately 2 h after the cell separation. Time code and the scale bars are at the bottom of the video.

## Data Availability

The data that support the findings of this study are available in the Materials and Methods, Results, and/or Supporting Information of this article.

## References

[1] M. J. Lohse , J. L. Benovic , J. Codina , M. G. Caron , and R. J. Lefkowitz , “β‐Arrestin: A Protein That Regulates β‐Adrenergic Receptor Function,” Science 248 (1990): 1547–1550. 10.1126/science.2163110 2163110

[2] S. K. Shenoy and R. J. Lefkowitz , “β‐Arrestin‐Mediated Receptor Trafficking and Signal Transduction,” Trends in Pharmacological Sciences 32 (2011): 521–533. 10.1016/j.tips.2011.05.002 PMC3159699 21680031

[3] R. J. Lefkowitz , K. Rajagopal , and E. J. Whalen , “New Roles for β‐Arrestins in Cell Signaling: Not Just for Seven‐Transmembrane Receptors,” Molecular Cell 24 (2006): 643–652. 10.1016/j.molcel.2006.11.007 17157248

[4] J. Wess , A.‐B. Oteng , O. Rivera‐Gonzalez , E. V. Gurevich , and V. V. Gurevich , “β‐ Arrestins: Structure, Function, Physiology, and Pharmacological Perspectives,” Pharmacological Reviews 75 (2023): 854–884. 10.1124/pharmrev.121.000302 PMC10441628 37028945

[5] L. Basso , R. Aboushousha , C. Y. Fan , et al., “TRPV1 Promotes Opioid Analgesia During Inflammation,” Science Signaling 12 (2019): eaav0711. 10.1126/scisignal.aav0711 30940767

[6] A. Hassan , M. Iftinca , D. Young , et al., “TRPV1 Activation Promotes β‐arrestin2 Interaction With the Ribosomal Biogenesis Machinery in the Nucleolus: Implications for p53 Regulation and Neurite Outgrowth,” International Journal of Molecular Sciences 22 (2021): 2280. 10.3390/ijms22052280 PMC7956682 33668926

[7] K. Xiao , D. B. McClatchy , A. K. Shukla , et al., “Functional Specialization of Beta‐Arrestin Interactions Revealed by Proteomic Analysis,” Proceedings of the National Academy of Sciences of the United States of America 104 (2007): 12011–12016. 10.1073/pnas.0704849104 PMC1913545 17620599

[8] H. Shankar , A. Michal , R. C. Kern , D. S. Kang , V. V. Gurevich , and J. L. Benovic , “Non‐Visual Arrestins Are Constitutively Associated With the Centrosome and Regulate Centrosome Function,” Journal of Biological Chemistry 285 (2010): 8316–8329. 10.1074/jbc.M109.062521 PMC2832982 20056609

[9] M. G. H. Scott , E. Le Rouzic , A. Périanin , et al., “Differential Nucleocytoplasmic Shuttling of β‐Arrestins. Characterization of a Leucine‐Rich Nuclear Export Signal in β‐arrestin2,” Journal of Biological Chemistry 277 (2002): 37693–37701. 10.1074/jbc.M207552200 12167659

[10] R. H. Oakley , S. A. Laporte , J. A. Holt , M. G. Caron , and L. S. Barak , “Differential Affinities of Visual Arrestin, Beta‐arrestin1, and Beta‐arrestin2 for G Protein‐Coupled Receptors Delineate Two Major Classes of Receptors,” Journal of Biological Chemistry 275 (2000): 17201–17210. 10.1074/jbc.M910348199 10748214

[11] P. Wang , Y. Wu , X. Ge , L. Ma , and G. Pei , “Subcellular Localization of Beta‐Arrestins Is Determined by Their Intact N Domain and the Nuclear Export Signal at the C Terminus,” Journal of Biological Chemistry 278 (2003): 11648–11653. 10.1074/jbc.M208109200 12538596

[12] F. Di Virgilio , D. Dal Ben , A. C. Sarti , A. L. Giuliani , and S. Falzoni , “The P2X7 Receptor in Infection and Inflammation,” Immunity 47 (2017): 15–31. 10.1016/j.immuni.2017.06.020 28723547

[13] J. Linden , F. Koch‐Nolte , and G. Dahl , “Purine Release, Metabolism, and Signaling in the Inflammatory Response,” Annual Review of Immunology 37 (2019): 325–347. 10.1146/annurev-immunol-051116-052406 30676821

[14] R. Lara , E. Adinolfi , C. A. Harwood , et al., “P2X7 in Cancer: From Molecular Mechanisms to Therapeutics,” Frontiers in Pharmacology 11 (2020): 793. 10.3389/fphar.2020.00793 PMC7287489 32581786

[15] P. Roussel , C. André , L. Comai , and D. Hernandez‐Verdun , “The rDNA Transcription Machinery Is Assembled During Mitosis in Active NORs and Absent in Inactive NORs,” Journal of Cell Biology 133 (1996): 235–246. 10.1083/jcb.133.2.235 PMC2120807 8609158

[16] F.‐M. Boisvert , S. van Koningsbruggen , J. Navascués , and A. I. Lamond , “The Multifunctional Nucleolus,” Nature Reviews. Molecular Cell Biology 8 (2007): 574–585. 10.1038/nrm2184 17519961

[17] S. Cankurtaran‐Sayar , K. Sayar , and M. Ugur , “P2X7 Receptor Activates Multiple Selective Dye‐Permeation Pathways in RAW 264.7 and Human Embryonic Kidney 293 Cells,” Molecular Pharmacology 76 (2009): 1323–1332. 10.1124/mol.109.059923 19749088

[18] P. Molinari , V. Vezzi , M. Sbraccia , et al., “Morphine‐Like Opiates Selectively Antagonize Receptor‐Arrestin Interactions,” Journal of Biological Chemistry 285 (2010): 12522–12535. 10.1074/jbc.M109.059410 PMC2857100 20189994

[19] M. T. Young , P. Pelegrin , and A. Surprenant , “Amino Acid Residues in the P2X7 Receptor That Mediate Differential Sensitivity to ATP and BzATP,” Molecular Pharmacology 71 (2007): 92–100. 10.1124/mol.106.030163 17032903

[20] O. Ugur and T. L. Z. Jones , “A Proline‐Rich Region and Nearby Cysteine Residues Target XLas to the Golgi Complex Region,” Molecular Biology of the Cell 11 (2000): 1421–1432. 10.1091/mbc.11.4.1421 PMC14856 10749939

[21] D. A. Groarke , S. Wilson , C. Krasel , and G. Milligan , “Visualization of Agonist‐Induced Association and Trafficking of Green Fluorescent Protein‐Tagged Forms of Both Beta‐Arrestin‐1 and the Thyrotropin‐Releasing Hormone Receptor‐1,” Journal of Biological Chemistry 274 (1999): 23263–23269. 10.1074/jbc.274.33.23263 10438501

[22] X. Song , D. Raman , E. V. Gurevich , S. A. Vishnivetskiy , and V. V. Gurevich , “Visual and Both Non‐Visual Arrestins in Their “Inactive” Conformation Bind JNK3 and Mdm2 and Relocalize Them From the Nucleus to the Cytoplasm,” Journal of Biological Chemistry 281 (2006): 21491–21499. 10.1074/jbc.M603659200 PMC2430869 16737965

[23] E. Muro , J. Gébrane‐Younès , A. Jobart‐Malfait , E. Louvet , P. Roussel , and D. Hernandez‐Verdun , “The Traffic of Proteins Between Nucleolar Organizer Regions and Prenucleolar Bodies Governs the Assembly of the Nucleolus at Exit of Mitosis,” Nucleus 1 (2010): 202–211. 10.4161/nucl.1.2.11334 PMC3030696 21326952

[24] A. K. L. Leung , D. Gerlich , G. Miller , et al., “Quantitative Kinetic Analysis of Nucleolar Breakdown and Reassembly During Mitosis in Live Human Cells,” Journal of Cell Biology 166 (2004): 787–800. 10.1083/jcb.200405013 PMC2172103 15353547

[25] D. Hernandez‐Verdun , “Assembly and Disassembly of the Nucleolus During the Cell Cycle,” Nucleus 2 (2011): 189–194. 10.4161/nucl.2.3.16246 PMC3149879 21818412

[26] L. Pecze , W. Blum , and B. Schwaller , “Mechanism of Capsaicin Receptor TRPV1‐Mediated Toxicity in Pain‐Sensing Neurons Focusing on the Effects of Na(+)/ca(2+) Fluxes and the ca(2+)‐Binding Protein Calretinin,” Biochimica et Biophysica Acta 1833 (2013): 1680–1691. 10.1016/j.bbamcr.2012.08.018 22982061

[27] M.‐K. Chung , A. D. Güler , and M. J. Caterina , “TRPV1 Shows Dynamic Ionic Selectivity During Agonist Stimulation,” Nature Neuroscience 11 (2008): 555–564. 10.1038/nn.2102 18391945

[28] J. A. Hiscox , “The Nucleolus – A Gateway to Viral Infection?,” Archives of Virology 147 (2002): 1077–1089. 10.1007/s00705-001-0792-0 PMC7087241 12111420

[29] T. E. Audas , M. D. Jacob , and S. Lee , “Immobilization of Proteins in the Nucleolus by Ribosomal Intergenic Spacer Noncoding RNA,” Molecular Cell 45 (2012): 147–157. 10.1016/j.molcel.2011.12.012 22284675

[30] M. D. Jacob , T. E. Audas , J. Uniacke , L. Trinkle‐Mulcahy , and S. Lee , “Environmental Cues Induce a Long Noncoding RNA–Dependent Remodeling of the Nucleolus,” Molecular Biology of the Cell 24 (2013): 2943–2953. 10.1091/mbc.E13-04-0223 PMC3771955 23904269

[31] P. J. Anderson , P. Xiao , Y.‐N. Zhong , et al., “β‐Arrestin Condensates Regulate G‐Protein‐Coupled Receptor Function,” Nature 655 (2026): 525–534. 10.1038/s41586-026-10539-y PMC13345904 42203872

[32] S. F. Banani , H. O. Lee , A. A. Hyman , and M. K. Rosen , “Biomolecular Condensates: Organizers of Cellular Biochemistry,” Nature Reviews. Molecular Cell Biology 18 (2017): 285–298. 10.1038/nrm.2017.7 PMC7434221 28225081

[33] P. Li , S. Banjade , H.‐C. Cheng , et al., “Phase Transitions in the Assembly of Multivalent Signalling Proteins,” Nature 483 (2012): 336–340. 10.1038/nature10879 PMC3343696 22398450

[34] J. Z. Zhang , T.‐W. Lu , L. M. Stolerman , et al., “Phase Separation of a PKA Regulatory Subunit Controls cAMP Compartmentation and Oncogenic Signaling,” Cell 182 (2020): 1531–1544. 10.1016/j.cell.2020.07.043 PMC7502557 32846158

[35] S. Hutten and R. H. Kehlenbach , “CRM1‐Mediated Nuclear Export: To the Pore and Beyond,” Trends in Cell Biology 17 (2007): 193–201. 10.1016/j.tcb.2007.02.003 17317185

